# Mitochondrial SIRT2-mediated CPT2 deacetylation prevents diabetic cardiomyopathy by impeding cardiac fatty acid oxidation

**DOI:** 10.7150/ijbs.102834

**Published:** 2025-01-01

**Authors:** Yaoyao Guo, Ziyin Zhang, Zheng Wen, Xiaonan Kang, Dan Wang, Lu Zhang, Mengke Cheng, Gang Yuan, Huihui Ren

**Affiliations:** 1 Division of Endocrinology, Department of Internal Medicine, Tongji Hospital, Tongji Medical College, Huazhong University of Science and Technology, Wuhan, China.; 2 Hubei Clinical Medical Research Center for Endocrinology and Metabolic Diseases, Hubei, China.; 3 Branch of National Clinical Research Center for Metabolic Diseases, Hubei, China.; 4 Division of Cardiology, Department of Internal Medicine, Tongji Hospital, Tongji Medical College, Huazhong University of Science and Technology, Wuhan, China.; 5 Hubei Key Laboratory of Genetics and Molecular Mechanisms of Cardiological Disorders, Hubei, China.

**Keywords:** diabetic cardiomyopathy, SIRT2, CPT2, fatty acid oxidation, deacetylation

## Abstract

Dysregulated energy metabolism, particularly lipid metabolism disorders, has been identified as a key factor in the development of diabetic cardiomyopathy (DCM). Sirtuin 2 (SIRT2) is a deacetylase involved in the regulation of metabolism and cellular energy homeostasis, yet its role in the progression of DCM remains unclear. We observed significantly reduced SIRT2 expression in DCM model mice. Cardiac-specific overexpression of SIRT2 protected mice from streptozotocin/high-fat diet (STZ/HFD)-induced insulin resistance (IR), cell apoptosis, and cardiac dysfunction, whereas its downregulation exacerbated these conditions. Moreover, we found that SIRT2 regulated cardiac lipid accumulation and fatty acid oxidation (FAO), and identified its localization in cardiac mitochondria. Mechanistically, we determined carnitine palmitoyltransferase 2 (CPT2) as a critical substrate of SIRT2, which is implicated in DCM. SIRT2-mediated deacetylation at K239 enhanced CPT2 ubiquitination, resulting in decreased protein stability and subsequent inhibition of FAO and reactive oxygen species (ROS) production. Taken together, these findings suggest that the SIRT2/CPT2 signaling pathway plays a crucial role in DCM progression.

## Introduction

The prevalence of diabetes mellitus (DM) has been continually increasing in the last decade, imposing a substantial burden on global public health. Cardiovascular disease is a lethal complication for diabetic patients and often causes heart failure 1, 2. Diabetic cardiomyopathy (DCM) has been identified as a significant health concern in the rapidly growing population of diabetic patients and is characterized in its early stages by diastolic abnormalities and later by clinical heart failure (HF) independent of recognized risk factors, including hypertension and coronary artery disease 3. The pathophysiology of DCM is complex and multifactorial and involves myocardial lipotoxicity, hyperglycemia, insulin resistance (IR), and oxidative stress 4, 5. Metabolic reprogramming is an important feature of DCM that is often accompanied by an increase in cardiac fatty acid (FA) uptake and fatty acid oxidation (FAO). In a diabetic heart, cardiac FA uptake is increased to compensate for the increased energetic demands of the heart, which cannot use glucose 6, 7. Greater cardiac FAO is accompanied by higher rates of oxygen consumption, resulting in increased reactive oxygen species (ROS) production 8. This shift in substrate use may play a central role in the pathogenesis of DCM. Moreover, FA can be converted to toxic intermediates, which may further increase mitochondrial damage and cardiac insulin resistance 6, 9. Thus, attenuation of cardiac fatty acid β-oxidation provides a potential therapeutic target for treating DCM.

SIRT2 (sirtuin 2), a specific sirtuin that can exist in both the cytosol and nucleus, is expressed in a wide range of tissues, such as the heart, adipose tissue, and brain 11, 12. Growing evidence suggests that SIRT2 participates in the regulation of gluconeogenesis, IR, and inflammation 13-15. Regarding lipid metabolism, SIRT2 deletion has been shown to improve the survival of memory T cells by enhancing FAO, which may contribute to superior antitumor immunity 16. In addition, SIRT2 can play an important role in maintaining cardiac homeostasis. It has recently been shown to exert a cardioprotective function in aged Ang II-infused mice through the deacetylation of the LKB1-AMPK pathway 17. Considering the close relationship between SIRT2 and metabolic homeostasis, as well as the protective role of SIRT2 in pathological cardiac hypertrophy, we explored the effect of SIRT2 on DCM and lipid metabolism.

Given the critical role of fatty acid metabolism in the pathophysiology of DCM, the function of carnitine palmitoyltransferase 2 (CPT2), an irreplaceable enzyme located in the inner mitochondrial membrane, becomes particularly important, as it is essential for the transport and oxidation of long-chain fatty acids within the mitochondria. These processes are key to maintaining cellular energy metabolism and heart function 18. CPT2-mediated FA metabolism has been proven to be involved in an array of cellular functions 19-21. Therefore, we explored the role of SIRT2 in a streptozotocin/high-fat diet (STZ/HFD)-induced mouse model. We hypothesize that SIRT2 through its deacetylation activity modulates the function of CPT2 and consequently fatty acid oxidation within cardiac mitochondria. In this study, we aim to assess the role of the SIRT2-CPT2 axis in DCM, potentially providing a therapeutic strategy for treating the condition.

## Methods

### Animal models

Male C57BL/6 mice aged 6-7 weeks were purchased from the Model Animal Research Center of Nanjing University (Jiangsu, China). In this study, only male mice were used because male mice are believed to exhibit more consistent biological characteristics in diabetes, and female mice may be influenced by hormonal cycles in metabolism 22. All the animals were raised in a room with a 12 h light/dark cycle and fed a standard chow diet. At the beginning of the experiment, all the mice were randomly divided into groups via the complete randomization method. After one week of adaptation, the mice received a normal chow diet (NCD, 10 kcal% fat, Research Diets, #D12450J) or a high-fat diet (HFD, 60 kcal% fat, Research Diets, #D12492). Moreover, the HFD group was intraperitoneally injected with STZ (30 mg/kg) for 3 consecutive days at 4 weeks, and HFD feeding was continued for up to 20 weeks. The control group of mice was injected with citrate buffer only. One week after STZ injection, the fasting blood glucose (FBG) levels of the mice were determined via an ACCU-Check Active digital glucometer. Mice with fasting blood glucose levels > 11.1 mmol/L were diagnosed with type II diabetes mellitus (T2D) 23. Different personnel performed different stages of the experiment to exclude bias due to subjective factors.

### Cell culture, treatment, and transfection

AC16, H9c2, and HEK293T cells were obtained from the American Type Culture Collection (ATCC, USA). SIRT2 knockout (KO) cells were created in AC16 cells background using the CRISPR-Cas9 system from Genechem (Shanghai, China). The target sequences of the gRNAs for human SIRT2 were 5'-ATACCCTGGAGCGAATAGCC-3'. The cells were cultured in Dulbecco's modified Eagle medium (DMEM) supplemented with 10% fetal bovine serum (FBS) (Gibco, #16140071) and 100 U/ml penicillin/100 mg/ml streptomycin in an atmosphere of 90% air and 10% CO_2_ at 37°C. To establish an *in vitro* model of DCM in AC16 cells, they were treated with 0.5 mM palmitic acid (PA) for 24 hours.

### Isolation and culture of neonatal rat cardiomyocytes (NRCMs)

NRCMs were isolated from the ventricles of Sprague-Dawley rats (0-3 days). Hearts were extracted from the body, placed in ice-cold Hanks' medium, and cut into pieces. The tissues were then digested with 0.075% collagenase II (Worthington, #LS004176) for 7 min at 37°C, and the digested cells were collected in DMEM containing 20% FBS. This process was repeated several times until the heart tissue was completely digested. The collected primary cells were passed through a cell strainer (200 mesh) and centrifuged (1200 × g, 10 minutes, 4°C). The cells were transferred to culture flasks and cultured for 2 hours to allow cardiac fibroblasts to adhere, while the cardiomyocytes were separated from the culture supernatant. Thereafter, the supernatants (cardiomyocytes) were plated in DMEM supplemented with 10% FBS. Primary cardiomyocytes at passage zero (not passaged) were used for experiments.

### Adeno-associated virus-mediated gene overexpression and knockdown

Recombinant adeno-associated viral 9 (AAV9)-cTnT (cardiac troponin T) gene transfer vectors bearing murine SIRT2 or CPT2 (AAV-SIRT2, AAV-CPT2) were constructed by Genechem (Shanghai, China) with a full-length SIRT2 or CPT2 complementary DNA (cDNA) coding sequence. For cardiac-specific SIRT2 or CPT2 overexpression in C57BL/6J male mice, the mice were injected via the tail vein with the adeno-associated virus (AAV) (1×10^11^ pfu, 100 µl) at the beginning of the experiment. AAV-GFP was injected into the mice as the corresponding control. For specific knockdown, a recombinant AAV-cTnT vector containing a short hairpin RNA targeting SIRT2 (AAV-shSIRT2) or CPT2 (AAV-shCPT2) was constructed by Genechem (Shanghai, China) and injected into mice via the jugular vein (1×10^11^ pfu, 100 µl). AAV-shRNA was injected into the mice as the corresponding control.

### Small-interfering RNA Transfection

All siRNAs were chemically synthesized by RiboBio (Guangdong, China). The siRNA sequences used were as follows: siSIRT2 (5'-CCTGCTCATCAACAAGGAGAA-3'); siCPT2 (5'-GACCCTGGTTTGATATGTA-3'). The cells were plated in 6-well plates and then transfected with 100 nM siRNA via Turbofect (Thermo Fischer Scientific, #R0531) following the manufacturer's instructions.

### Lentivirus infection

A recombinant SIRT2 lentivirus was constructed by Genechem (Shanghai, China). To induce the overexpression of SIRT2, cells were infected with control lentivirus or recombinant SIRT2 lentivirus at 10 M.O.I. for 48 hours in growth medium.

### Plasmid constructs

To obtain a vector overexpressing human SIRT2, a SIRT2 (NM_012237.3) expression vector with a C-terminal HA tag was constructed by ligating the corresponding full-length cDNA into a pCMV-C-HA vector (Beyotime, D2639). The human CPT2 (NM_000098.3) expression vector with an N-terminal FLAG tag was constructed by inserting FLAG-CPT2 cDNA into pcDNA3.1(+). SIRT2 or CPT2 mutants were generated via a site-directed mutagenesis kit (TransGen Biotech, #FM111-01). MTS-SIRT2 plasmid was constructed by PAIVIBIO (Wuhan, China). The sequences of primers used in this study are listed in Table S1.

### Blood glucose measurement, intraperitoneal glucose tolerance test (IPGTT), and intraperitoneal insulin tolerance test (IPITT)

For fasting blood glucose, the mice were fasted for 12 h, and then the body weight and blood glucose level were measured from the tip of the tail with an ACCU-Check Active digital glucometer. For IPGTTs, the mice were subjected to i.p. injection of 1 g/kg glucose after 12 h of fasting. Blood glucose was detected at 0, 15, 30, 60, and 120 minutes after injection. For the IPITTs, the mice were subjected to i.p. injection of 0.75 U/kg insulin after 6 h of fasting. Blood glucose was detected at 0, 15, 30, 60, and 120 minutes after injection. The glucose and insulin tolerance levels were reflected by AUCs 24.

### Biochemistry assay

The levels of heart free fatty acids were measured via a colorimetric diagnostic kit (Jiancheng Bioengineering Institute, #A042-2-1) according to the manufacturer's instructions. ELISA kits for TNF-α (Abcam, #ab208348) and IL-6 (Proteintech, #KE10091) were used to measure the levels of TNF-α and IL-6 in mouse plasma. All the operation steps were carried out according to the manual.

### Echocardiography analysis

Under anesthesia with inhaled 1.5% isoflurane, echocardiography was performed via a high-resolution imaging system with a VisualSonics Vevo 770 ultrasound biomicroscope (VisualSonics Inc.) with a 30-MHz linear array ultrasound transducer. After stabilization, the data were continuously recorded. Heart rate (HR), left ventricular end-systolic diameter (LVESD), left ventricular end-diastolic diameter (LVEDD), left ventricle (LV) internal dimension in diastole (LVID, d) and systole (LVID, s) were measured. The left ventricular ejection fraction (LVEF) and left ventricular fraction shortening (LVFS) were calculated via computer algorithms 25. The early (E) and late (A) diastolic mitral flow velocities were measured via Doppler echocardiography, and the E/A ratio was calculated. The E/A ratio was measured to evaluate diastolic function. Finally, all the mice were sacrificed for further studies after echocardiography analysis.

### Histological analysis

The heart tissues were fixed in 4% paraformaldehyde, embedded in paraffin, and sectioned into 5 µm slices. H&E staining was used to determine the cardiomyopathy cross-sectional areas and morphology. The degree of collagen deposition was detected via Masson's trichrome staining. Lipid droplet (LD) accumulation in the heart was analyzed via Oil Red O (ORO) staining. Immunohistochemistry was performed with an anti-SIRT2 antibody (Abcam, #ab211033). Immunoreactivity was detected with diaminobenzidine (DAB).

### TUNEL staining

Cardiac apoptosis was determined via TUNEL staining according to the manufacturer's instructions (Roche, #12156792910). Staining with DAPI and TUNEL was performed to confirm the presence of apoptotic nuclei. The slices were observed under a fluorescence microscope, and the percentage of TUNEL-positive cells was calculated.

### Immunofluorescence staining

AC16 cells were cultured on glass coverslips and stained with 2 μM BODIPY 493/503 dye (Amgicam, #ajci70160) for 30 min or incubated with 300 nM MitoTracker Green dye (Thermo Fisher Scientific, #M7514) for 20 min at 37°C. After washing, the sections were stained with DAPI and covered with glass coverslips. For colocalization, the cells were fixed with 4% paraformaldehyde for 30 min, permeabilized with 0.2% Triton X-100 for 15 min, and then washed twice with PBS. After being blocked with PBS containing 5% FBS and 1% BSA, the cells were incubated with anti-SIRT2 (Abcam, #ab211033) or anti-CPT2 (Abcam, #ab110293) antibodies in PBS at 4°C overnight. The following day, after three washes in PBS with Tween 20 (PBST), the cells were incubated with secondary antibodies in PBS at room temperature for 2 h and rinsed with PBST. Then, the slides were mounted with mounting media containing DAPI.

### Electron microscopy

Heart tissues were prefixed with 2.5% glutaraldehyde in 0.1 M sodium cacodylate buffer (pH 7.4) for 2 h (4°C) and then postfixed with 1% osmium tetroxide (1 h). After dehydration, the samples were embedded in epoxy resin and cut into ultrathin sections (60 nm). The sections were examined with a JEM-1400 transmission electron microscope (JEOL, Japan).

### Mitochondrial fatty acid oxidation

The rate of heart tissue FAO was measured via the Fatty Acid Oxidation Assay Kit (AssayGenie, Dublin, Ireland). The assay was performed according to the manufacturer's instructions, and the colorimetric reaction was read at 492 nm on a microplate reader (BIO-TEK instruments Inc., USA).

### Seahorse assay

Cellular oxygen consumption rates (OCRs, Agilent, 103015-100) were measured in real time via an XF96 Extracellular Flux Analyzer (Agilent, USA). AC16 cells were transferred to a seahorse cell culture plate and then treated with or without SIRT2/siSIRT2. After that, the cells were pretreated with palmitate-BSA (500 μM) or BSA for 24 h. For the mitochondrial stress test, oligomycin (1.5 µM), FCCP (1 µM), and rotenone/antimycin (0.5 µM) were used, and the OCR was measured. For the fatty acid oxidation assay (Agilent, 103693-100), the cells were incubated overnight in seahorse substrate-limited medium supplemented with 0.5 mM glucose, 1 mM glutamine, 0.5 mM carnitine (MedChemExpress, #HY-B0399) and 1% FBS. Before the assay, the cells were replenished with FAO assay medium and incubated for 30 min. After that, the cells were pretreated with etomoxir (4 μM, MedChemTronica, #HY-502002), palmitate (500 μM) or BSA for another 20 min. The OCR was measured following the sequential addition of oligomycin (1.5 µM), FCCP (1 µM), and rotenone/antimycin (0.5 µM). The OCR was analyzed and normalized to the amount of total protein 26.

### Mitochondrial fractionation and submitochondrial localization assay

The mitochondria isolation assay was carried out via a mitochondria isolation kit (Beyotime Institute of Biotechnology, #C3601, #C3606). Briefly, AC16 cells, NRCMs, and heart tissues were collected in mitochondria isolation buffer (provided in the kit) on ice for 10 min and homogenized with 30 strokes of a homogenizer. The homogenate was centrifuged at 600 × g for 10 min at 4°C to separate the organelles from the cellular debris. Then, the supernatant was centrifuged again at 12,000 × g for 30 min at 4°C. Samples of cytosolic (supernatant) and mitochondrial (deposition) fractions were dissolved in lysis buffer, and the proteins were subjected to western blotting 12. To further investigate the localization of SIRT2 in the mitochondria (mitochondrial membrane (OMM), inner mitochondrial membrane (IMS), and matrix), 1 mg/mL proteinase K (Solarbio, #P9460), with or without 20% Triton X-100 (Solarbio, T8200), was added to the isolated mitochondrial fractions (100 μg per treatment), which were incubated on ice for 1 h and then terminated with PMSF. The proteins were subjected to western blotting.

### CCK8

CCK-8 (MedChemExpress, #HY-K0301) was used to detect cardiomyocyte viability. The cells were seeded into 96-well plates at 3000 cells/well and treated or cultured accordingly, followed by the addition of 10 μl of CCK-8 solution. After 2 h, the OD value at 450 nm was read to measure the viability of the cells.

### Cell apoptosis assays

To evaluate cell apoptosis, AC16 cells were incubated with an Annexin Apoptosis Detection Kit (BD Pharmingen™, #556547). Briefly, the cells were resuspended in binding buffer and incubated with fluorescein isothiocyanate (FITC)-conjugated annexin V and propidium iodide (PI) according to the manufacturer's protocol. After incubation, the cells were analyzed with a FACStar Plus flow cytometer (Beckman Coulter, USA), and Annexin-V-positive and PI-positive cells were identified as apoptotic cells.

### ROS detection

Intracellular ROS levels were detected by DCFH-DA (Beyotime Institute of Biotechnology, #S0033S). The cells were incubated with 10 μM DCFH-DA in serum-free DMEM for 30 min at 37°C in the dark. After the cells were washed twice with cold PBS, the fluorescence intensity of the cells was measured immediately with a FACStar Plus flow cytometer (Beckman Coulter, USA).

### Western blot analysis

All protein samples were separated on SDS-PAGE gels and then transferred to PVDF membranes (polyvinylidene difluoride, Millipore, #IPVH00010). After being blocked with 5% skim milk for 2 h at room temperature, the membranes were incubated overnight at 4°C with antibodies. After the blots were washed to remove excess primary antibody, they were incubated for 2 h with peroxidase-conjugated secondary antibodies. The antibodies used in this study are listed in Table S2. Antibody binding was detected via a chemiluminescence system (Bio-Ras Laboratories, USA), and the band intensity was quantified via the ImageJ program.

### Immunoprecipitation

AC16 cells or HEK293T cells were lysed in lysis buffer (20 mM Tris-HCl, pH 8.0, 100 mM NaCl, 1 mM EDTA, and 0.5% NP-40) containing a protease on ice for 30 minutes. The cell homogenates were then centrifuged at 13,000 × g for 20 minutes. Then, 2 μl of primary antibody (anti-SIRT2, anti-CPT2, anti-IgG, anti-Flag, or anti-HA) was added to the supernatant, and the mixture was incubated with rocking at 4°C overnight. The following day, 30 μl of protein A/G magnetic beads (MCE, #HY-K0202) were added, and the sample was incubated at 4°C for 3 h. The beads were washed 5 times with the corresponding lysis buffer, boiled with 1✕buffer, and analyzed by western blotting 27.

### GST pull-down

The GST-SIRT2 fusion protein was purchased from Proteintech (#Ag7556), while Myc/Flag-CPT2 was purchased from ORIGENE (#TP300484), and the GST-WT fusion protein was purchased from ABclonal (#RPT0001LQ). Equal amounts of GST fusion proteins (conjugated on beads) were incubated with 1 ml of cell lysate (NP40 containing Myc/Flag-CPT2) overnight with rotation at 4°C and then washed three times with NP40. The protein was boiled in 1× loading buffer and analyzed by western blotting with the indicated antibodies 28.

### Real-time quantitative PCR

Total RNA was extracted from the heart tissues of the mice and AC16 cells with TRIzol Reagent (Thermo Fischer Scientific, #15596018), reverse transcribed into cDNA and used to conduct real-time quantitative PCR (qRT-PCR) assays for genes. β-actin was used as an internal control, and the PCR experiments were repeated three times. The fold change (2^-ΔΔCT^) represents the relative gene expression level. The sequences of primers used in this study are listed in Table S3.

### TMT-based proteomics

AC16 cells transfected with lenti-vector-GFP or lenti-SIRT2-GFP for 48 h were incubated with PA for 24 h. For proteomic quantification, suitable samples (>2 × 10^7^ cells) were harvested and frozen in liquid nitrogen for protein extraction. Each group had at least three independent biological replicates. TMT-based proteomics analysis was performed at PTM Bio (Hangzhou, China). Then, via enrichment analysis and protein-protein interaction network analysis, the protein network data were visualized via Cytoscape (v.3.5.1).

### Mass spectrometry

To identify the acetylation site of CPT2, flag-CPT2 was transfected into AC16 cells, and protein samples were separated via SDS-PAGE. After Coomassie blue staining, the gel strip containing the protein of interest was cut out, then purified and resolved. Following in-gel digestion, the CPT2 peptide mixture was analyzed via HPLC-MS/MS at PTM Bio (Hangzhou, China). The data were analyzed via Mascot v.2.1 software (Matrix Science, USA).

### Statistical analysis

All the data are shown as the means ± SDs unless otherwise noted. Unpaired Student's t test was used to compare two independent groups, one-way ANOVA was used to analyze a dependent variable with more than two independent samples, and two-way ANOVA was used to evaluate two independent variables with more than two independent samples. If the P value is less than 0.05, there is a difference between the experimental group and the control group. P values are represented as follows: ^#^P < 0.05;^ ##^P < 0.01;^ ###^P < 0.001; ^####^P < 0.0001; *P < 0.05; **P < 0.01; ***P < 0.001; ****P < 0.0001; ns, not significant. The data represent the mean ± SD. All the statistical tests were performed via GraphPad Prism (v9.0) unless otherwise stated.

## Results

### Cardiac SIRT2 expression is reduced in diabetic cardiomyopathy mice and cells

**Figure 1:**
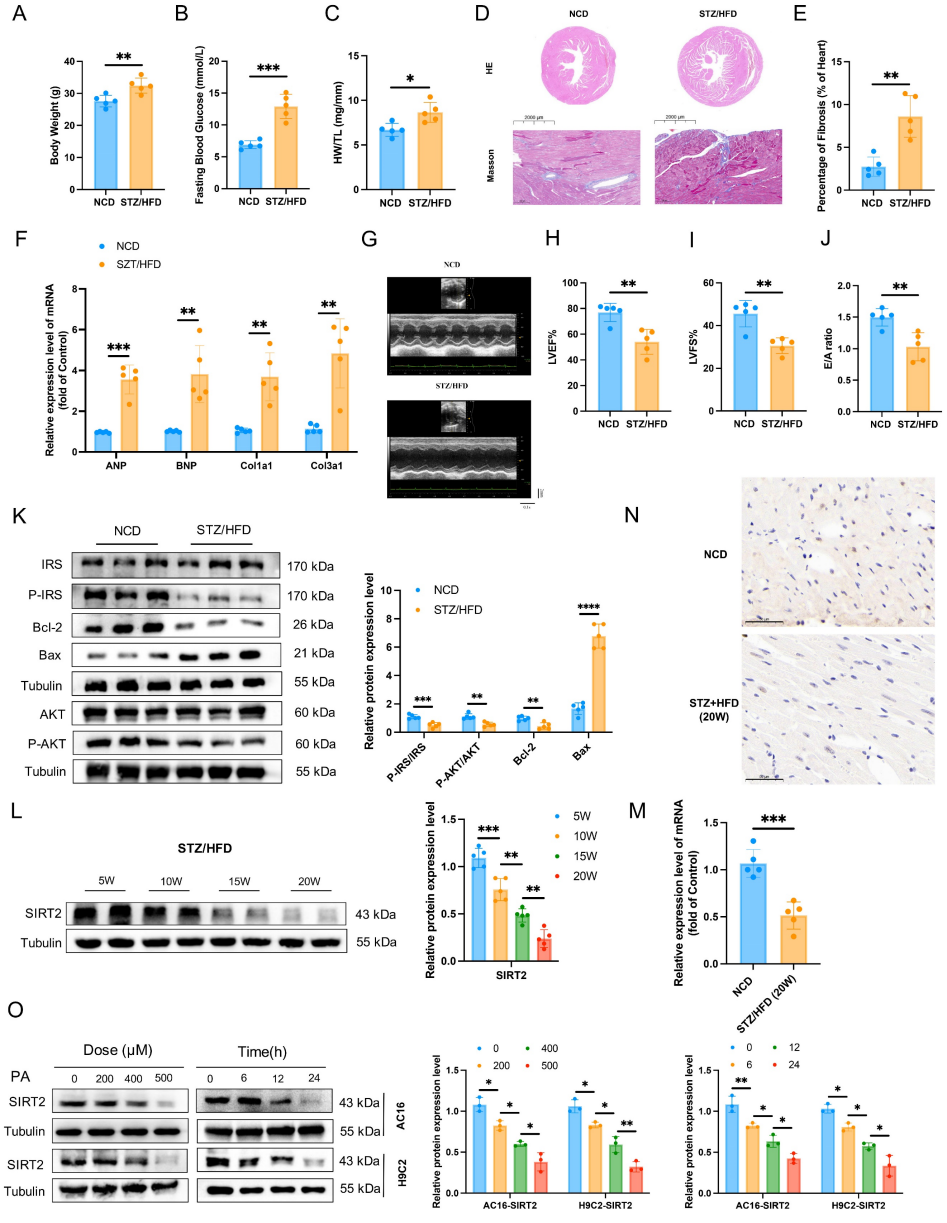
** Cardiac SIRT2 expression is reduced in DCM male mice.** C57BL/6J male mice were fed a NCD or STZ/HFD for 20 weeks (NCD, n=5; STZ/HFD, n=5). **(A)** Representative body weights of the mice.** (B)** Representative FBG data of the mice. **(C)** Representative HW/TL of the mice. **(D-E)** Representative images of heart sections stained with HE (top) and Masson (bottom) are shown (D). Quantification of the Masson staining area in heart sections is shown (E). Scale bars, 2000 μm (HE) and 100 μm (Masson). **(F)** Hypertrophic gene and fibrotic gene mRNA expression in DCM mice was tested by qRT-PCR. **(G)** Representative images of echocardiograms. **(H-I)** Percentages of LVEF (H) and LVFS (I) in the mice. **(J)** The E/A ratio in the mice.** (K)** Protein levels of the insulin receptor signaling pathway, the apoptosis gene Bax, and the anti-apoptotic gene Bcl2 in mouse heart tissue were measured. **(L)** The protein level of SIRT2 in heart lysates was measured. **(M)** Relative mRNA levels of SIRT2 in the heart tissue of the NCD-fed and STZ/HFD-fed mice. **(N)** Immunohistology staining of SIRT2 in heart tissue sections from the NCD-fed and STZ/HFD (20 W)-fed mice. Scale bars, 50 μm. **(O)** AC16 and H9c2 cells were treated with 0 μM, 200 μM, 400 μM, or 500 μM PA for 24 hours. AC16 and H9c2 cells were treated with 500 μM PA for the indicated times. The SIRT2 protein expression level was measured via western blotting (n=3). The data are presented as the mean ± SD. *p<0.05; **p<0.01; ***p<0.001. Statistical analysis was performed via unpaired Student's t test and one-way ANOVA.

To investigate the potential role of SIRT2 in DCM, we first induced a type II diabetic mouse model by feeding C57BL/6 mice a HFD combined with three boluses of STZ for sequential observation for up to 20 weeks (Supplementary Fig. 1A). STZ/HFD challenge induced diabetes in the mice (Supplementary Fig. 1B, C). After 20 weeks, the body weights and FBG levels of the STZ/HFD-induced diabetic mice were significantly different from those of the NCD-fed mice (Fig. 1A, B). Furthermore, STZ/HFD-induced diabetic mice presented obvious increases in the heart weight-to-tibia length ratio (HW/TL) (Fig. 1C), myocardial hypertrophy, and fibrosis (Fig. 1D, E). Consistently, the qRT-PCR results revealed that these pathological phenotypes were accompanied by the upregulation of hypertrophic genes and fibrotic genes, including atrial natriuretic polypeptide (*Anp*), brain natriuretic peptide (*Bnp*), collagen 1a1 (*Col1a1*), and collagen 3a1 (*Col3a1*) (Fig. 1F). In STZ/HFD-induced diabetic mice, echocardiographic evaluation revealed systolic and diastolic dysfunction, as manifested by slightly increased LVID (Fig. 1G) and decreases in EF (Fig. 1H), FS (Fig. 1I), and the E/A ratio (Fig. 1J). TUNEL staining analysis revealed that the number of apoptotic cells was significantly greater in hearts from the STZ/HFD group than in those from the NCD group (Supplementary Fig. 1D). The STZ/HFD-induced mice presented increased expression of the proapoptotic gene Bax and decreased expression of the antiapoptotic gene Bcl2 in heart tissue (Fig. 1K). Moreover, STZ/HFD-induced diabetic mice presented obviously impaired insulin signaling (Fig. 1K). All these characteristics observed in STZ/HFD-induced diabetic mice are consistent with the pathophysiology of DCM. Therefore, STZ/HFD-induced diabetic mice might constitute a useful model of experimental DCM. Considering that the roles of SIRT3 and SIRT5 in diabetic cardiomyopathy have been validated 23, 28, we assessed the expression patterns of SIRT2 and SIRT4 in heart tissues. There was a significant decrease in the mRNA and protein levels of SIRT2, but not SIRT4, in the cardiac tissues of the STZ/HFD-fed mice compared with those of the NCD control mice (Fig. 1L, M and Supplementary Fig. 2), so we focused on investigating the potential role of SIRT2 in the regulation of DCM. Notably, the protein levels of SIRT2 gradually decreased in the heart from 5-20 weeks (Fig. 1L and Supplementary Fig. 2). Moreover, immunohistochemical staining revealed that SIRT2 expression was significantly lower in the hearts of STZ/HFD-induced diabetic mice than in those of control mice (Fig. 1N). We subsequently employed PA to establish a cellular model mimicking lipotoxicity in diabetic cardiomyopathy. Next, we investigated the expression of SIRT2 in both AC16 and H9c2 cells. A CCK-8 assay revealed that AC16 cell viability decreased with increasing concentrations of PA from 0 µM to 700 µM (Supplementary Fig. 3). On the basis of our results and a literature search 23, 500 µM PA was selected as the optimum dose for further studies. A significant reduction in SIRT2 expression occurred with PA in a time-dependent and dose-dependent manner (Fig. 1O). Under PA stimulation, cells presented increased insulin resistance, cell apoptosis, hypertrophy, and fibrosis (Supplementary Fig. 4). Collectively, these data indicate that cardiac SIRT2 expression obviously decreases in DCM, which may play a key role in the pathogenesis of this disease.

### Overexpression of cardiac SIRT2 relieves STZ/HFD-induced insulin resistance, cell apoptosis, and cardiac dysfunction

**Figure 2:**
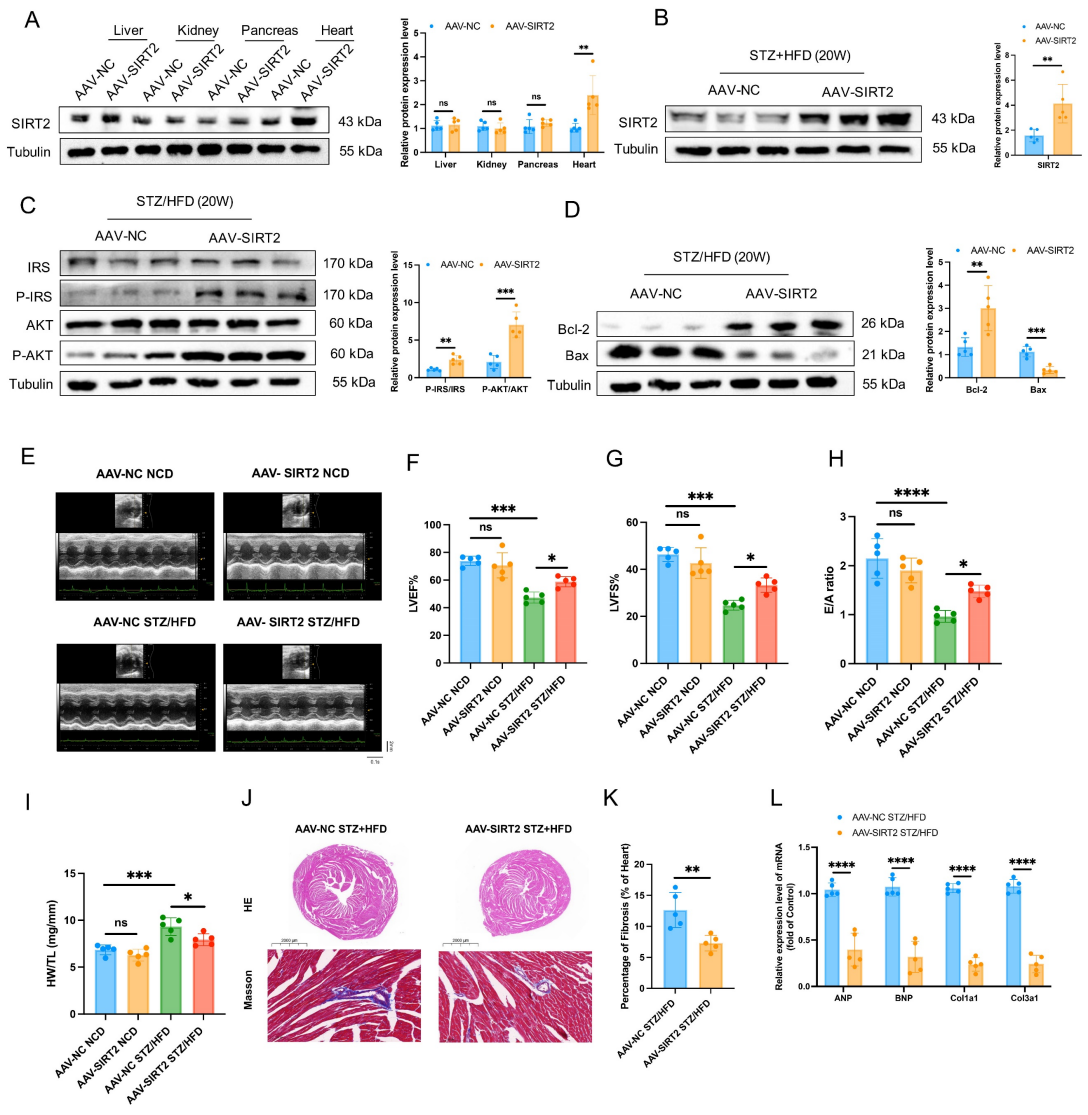
** Cardiac SIRT2 overexpression protects against insulin resistance, cell apoptosis, and cardiac dysfunction in DCM male mice. (A)** C57BL/6J male mice were fed a NCD or STZ/HFD for 20 weeks after being injected with 1×10^11^ pfu AAV-NC (n=5) or AAV-SIRT2 (n=5) through the tail vein. The protein expression of SIRT2 in the liver, kidney, pancreas, and heart. **(B)** The protein expression of SIRT2 in mouse heart tissue after virus injection and induction by STZ/HFD. **(C)** Protein expression of insulin receptor signaling components in mouse heart tissue. **(D)** Protein expression of the apoptosis gene Bax and anti-apoptotic gene Bcl2 in mouse heart tissue. **(E)** Representative images of echocardiograms. **(F-H)** Percentages of LVEF (F), LVFS (G), and E/A (H). **(I)** Representative HW/TL of the mice described in (A). **(J-K)** Representative images of heart sections stained with HE (top) and Masson (bottom) are shown (J). Quantification of the Masson staining area in heart sections is shown (K). Scale bars, 2000 μm (HE), and 100 μm (Masson). **(L)** Hypertrophic gene and fibrotic gene mRNA expression in mouse heart tissues was tested via qRT-PCR. The data are presented as the mean ± SD. *p<0.05; **p<0.01; ***p<0.001; ****p<0.0001; ns indicates no significance. Statistical analysis was performed via one-way ANOVA and unpaired Student's t test.

Given that cardiac SIRT2 expression is reduced in DCM models, we next investigated the pathophysiological role of SIRT2 in the heart. Cardiomyocyte-specific SIRT2-overexpressing mice were generated from commercially available AAV (Fig. 2A and Supplementary Fig. 5A). To this end, AAV-SIRT2 and AAV-NC mice were subjected to STZ/HFD challenges or NCD. As shown in Fig. 2B, compared with AAV-NC, AAV-SIRT2 increased SIRT2 expression in the hearts of STZ/HFD-induced mice. Under STZ/HFD challenge, the mRNA levels of inflammatory genes and plasma inflammatory factors were reduced in AAV-SIRT2 mouse hearts (Supplementary Fig. 6A-C). Our subsequent research revealed that SIRT2 overexpression in STZ/HFD-treated mice not only impacted cardiac inflammation but also further affected peripheral glycolipid metabolism, as indicated by reduced body weight (Supplementary Fig. 6D), lower FBG levels (Supplementary Fig. 6E), and improved glucose tolerance and insulin sensitivity (Supplementary Fig. 6F, G). Moreover, insulin signaling was improved in the hearts of the AAV-SIRT2 STZ/HFD model mice, as reflected by increased levels of phosphorylated IRS and AKT (Fig. 2C). Moreover, AAV-NC STZ/HFD-fed mice exhibited a robust increase in cell apoptosis, whereas this change was attenuated in AAV-SIRT2 STZ/HFD-fed mice (Fig. 2D and Supplementary Fig. 7A).

To understand the cardiac phenotypes, we conducted echocardiographic assessments in all 4 groups of experimental mice. There were significant differences between the STZ/HFD and NCD groups. Compared with AAV-NC STZ/HFD-fed mice, AAV-SIRT2 STZ/HFD-fed mice presented improved systolic and diastolic function, manifested by slightly reduced LVID and increased EF, FS, and E/A ratios (Fig. 2E-H). However, the HR was similar among all the experimental groups (Supplementary Fig. 6H). In addition, the AAV-SIRT2 STZ/HFD-fed mice presented a reduction in HW/TL, cardiac hypertrophy, and cardiac fibrosis (Fig. 2I-L). Similarly, in AC16 and H9c2 cells, overexpressing SIRT2 via transfection of a plasmid carrying the SIRT2 gene reduced insulin resistance, cell apoptosis, hypertrophy, and fibrosis (Supplementary Fig. 8A-C and Supplementary Fig. 9A). Taken together, these results show that cardiac SIRT2 overexpression blunts insulin resistance, cell apoptosis, and cardiac dysfunction in DCM, supporting the protective role of SIRT2 in DCM.

### Deficiency of cardiac SIRT2 exacerbates STZ/HFD-induced insulin resistance, cell apoptosis, and cardiac dysfunction

**Figure 3:**
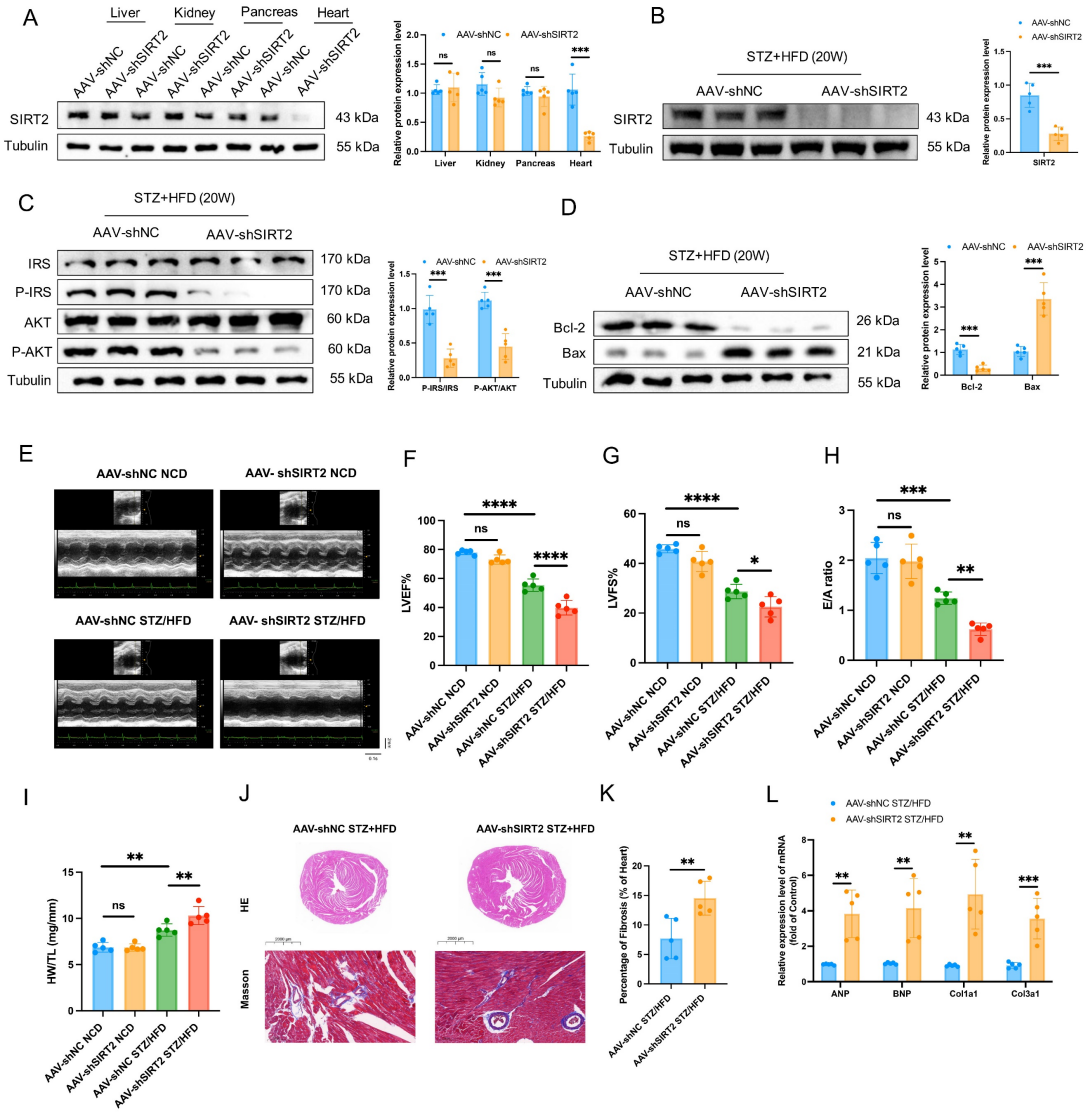
** Cardiac SIRT2 deficiency exacerbates insulin resistance, cell apoptosis, and cardiac dysfunction in DCM male mice. (A)** C57BL/6J male mice were fed a NCD or STZ/HFD for 20 weeks after being injected with 1×10^11^ pfu AAV-shNC (n=5) or AAV-shSIRT2 (n=5) through the tail vein. The protein expression of SIRT2 in the liver, kidney, pancreas, and heart. **(B)** The protein expression of SIRT2 in mouse heart tissue after virus injection and induction by STZ/HFD. **(C)** Protein expression of insulin receptor signaling components in mouse heart tissue. **(D)** Protein expression of the apoptosis gene Bax and anti-apoptotic gene Bcl2 in mouse heart tissue. **(E)** Representative images of echocardiograms. **(F-H)** Percentages of LVEF (F), LVFS (G), and E/A (H). **(I)** Representative HW/TL of the mice described in (A). **(J-K)** Representative images of heart sections stained with HE (top) and Masson (bottom) are shown (J). Quantification of the Masson staining area in heart sections is shown (K). Scale bars, 2000 μm (HE), and 100 μm (Masson). **(L)** Hypertrophic gene and fibrotic gene mRNA expression in mouse heart tissues was tested via qRT-PCR. The data are presented as the mean ± SD. *p<0.05; **p<0.01; ***p<0.001; ****p<0.0001; ns indicates no significance. Statistical analysis was performed via one-way ANOVA and unpaired Student's t test.

The opposite phenotypes were observed in AAV-mediated, heart-specific SIRT2-deficient mice (Fig. 3A). AAV-shSIRT2 STZ/HFD-fed mice (Fig. 3B and Supplementary Fig. 5B) exhibited aggravated inflammation (Supplementary Fig. 6I-K), increased body weight (Supplementary Fig. 6L), and elevated FBG levels (Supplementary Fig. 6M). Additionally, impaired glucose tolerance (Supplementary Fig. 6N), insulin tolerance (Supplementary Fig. 6O), disrupted insulin signaling (Fig. 3C) and increased cell apoptosis (Fig. 3D and Supplementary Fig. 7B) were evident in the AAV-shSIRT2-induced STZ/HFD-fed mice compared with the AAV-shNC-induced STZ/HFD-fed mice. Echocardiographic evaluation revealed that the LVID, EF, FS, and E/A ratio were further aggravated in the AAV-shSIRT2 STZ/HFD-fed mice (Fig. 3E-H), but there was no difference in the HR (Supplementary Fig. 6P). Additionally, the AAV-shSIRT2-induced STZ/HFD-induced mice presented more severe cardiac hypertrophy and cardiac fibrosis (Fig. 3I-L). Finally, SIRT2 silencing via siRNA in AC16 and H9c2 cells enhanced insulin resistance, cell apoptosis, hypertrophy, and fibrosis (Supplementary Fig. 8D-F and Supplementary Fig. 9B). These results indicate that cardiac SIRT2 deficiency specifically aggravates insulin resistance, cell apoptosis, and cardiac dysfunction in DCM.

### SIRT2 orchestrates cardiac fatty acid metabolism

**Figure 4:**
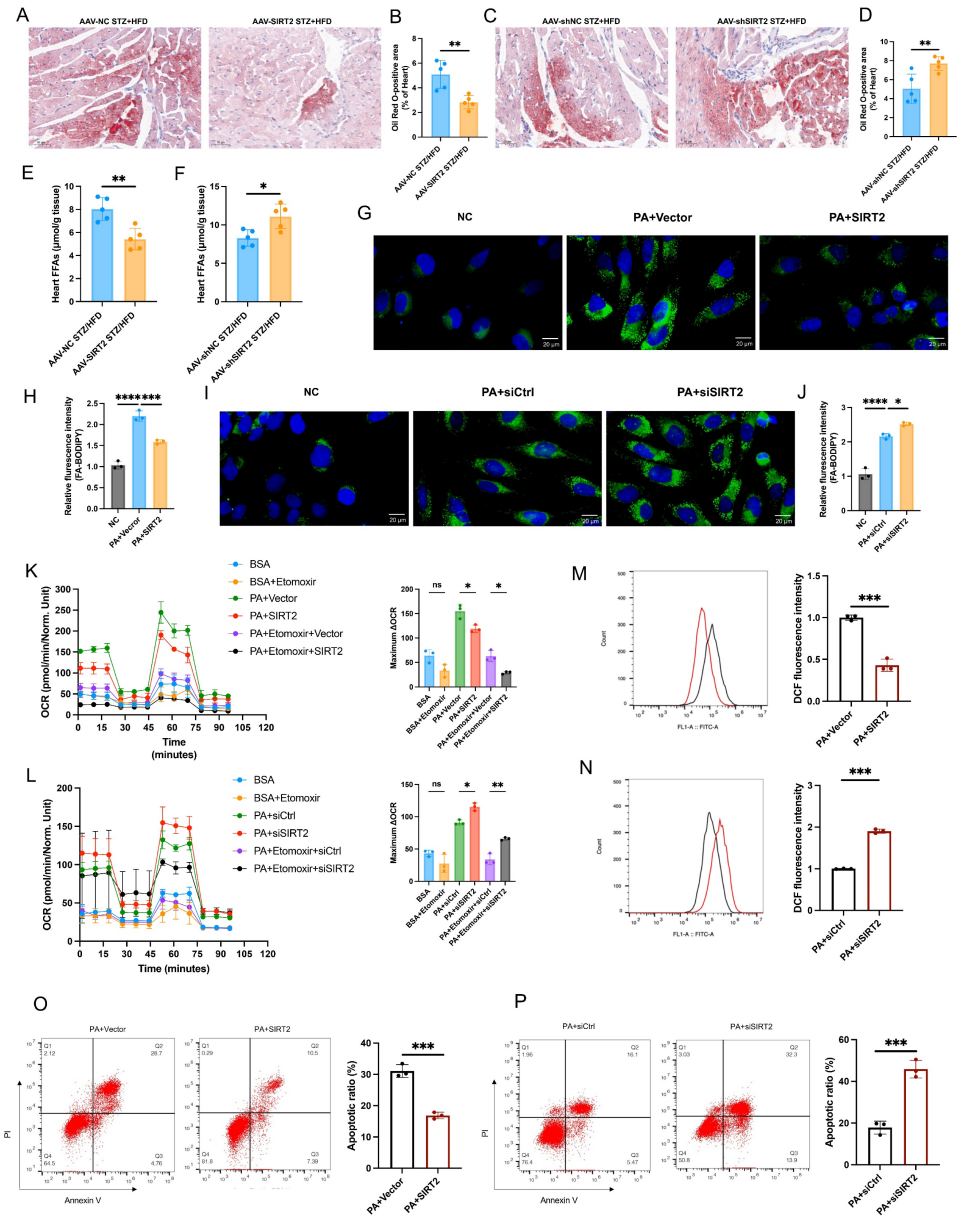
** SIRT2 improves fatty acid metabolism. (A-D)** Representative images of heart sections stained with Oil Red O are shown (A, C). Quantification of the Oil Red O-positive area in heart sections is shown (B, D). Scale bars, 50 μm. (n=5). **(E-F)** Heart FFA levels in cardiac-specific SIRT2-overexpressing/deficient mice (n=5). **(G-J)** Representative images and quantitative analysis of BODIPY 493/503 fluorescent dye staining of neutral lipids in cells. Scale bars, 20 μm. **(K-L)** FAO of AC16 cells was examined via the Seahorse assay (n=3).** (M-N)** ROS generation in cells was examined by flow cytometry after DHE probe treatment (n=3). **(O-P)** Apoptosis of AC16 cells was measured via flow cytometry with Annexin V and PI staining (n=3). The data are presented as the mean ± SD. *p<0.05; **p<0.01; ***p<0.001; ****p<0.0001; ns indicates no significance. Statistical analysis was performed via one-way ANOVA and unpaired Student's t test.

Cardiac lipotoxicity plays a pivotal role in the pathogenesis of DCM 29, 30. Under DCM, the heart relies mainly on free fatty acids (FFAs) for its energy needs, leading to increased mitochondrial oxidative stress and lipid peroxidation 31, 32. We directly quantified FAO and ATP levels in heart tissue and found that the overexpression of SIRT2 could slightly reduce FAO and ATP levels in cardiac tissue, whereas FAO and ATP levels were increased in the context of SIRT2 deficiency (Supplementary Fig. 10A, B, and see below). In addition, Oil Red O staining revealed that cardiac lipid droplet (LD) accumulation was significantly lower in the AAV-SIRT2 STZ/HFD group than in the control group; however, the AAV-shSIRT2 STZ/HFD group presented increased LD accumulation (Fig. 4A-D). Similarly, compared with control mice, AAV-SIRT2 STZ/HFD-fed mice presented significantly lower levels of heart FFAs, but AAV-shSIRT2 STZ/HFD-fed mice presented increased levels of FFAs (Fig. 4E, F). To further verify the effect of SIRT2 on FA metabolism, we conducted *in vitro* experiments using a fluorescent palmitate analog to detect LD accumulation. We observed that SIRT2 overexpression decreased the accumulation of LDs in AC16s, whereas SIRT2 knockdown increased LD accumulation (Fig. 4G-J). To quantify metabolic changes, we performed *in vitro* experiments in which cardiomyocytes were treated with the vector/SIRT2 plasmid or siCtrl/siSIRT2 and then assessed for 24 h of mitochondrial stress in the presence or absence of PA (500 μmol/L). FFA requires more oxygen for β-oxidation, and the OCR is an indicator of substrate utilization in cells 8. We found that PA treatment increased the maximal respiratory and ATP production. SIRT2 overexpression significantly reduced the maximal respiratory capacity, whereas SIRT2 knockdown resulted in the opposite phenotype (Supplementary Fig. 10C, D). Moreover, to assess FAO in cardiomyocytes, we used a palmitic acid oxidative stress assay. As expected, in diabetic cardiomyocytes overexpressing SIRT2, the maximum ΔOCR significantly decreased, whereas silencing SIRT2 increased the maximum ΔOCR (Fig. 4K, L). Additionally, excessive FAO rates contribute to ROS production, which can decrease cardiac efficiency. Therefore, we conducted further investigations in AC16 cells. The results revealed that overexpression of SIRT2 led to a reduction in cellular ROS levels and apoptosis (Fig. 4M, O), whereas silencing SIRT2 increased ROS levels and apoptosis (Fig. 4N, P). Overall, these findings demonstrate the involvement of SIRT2 in regulating fatty acid metabolism in the heart, and limiting FAO (despite a modest reduction in ATP) could protect cardiac cells by alleviating metabolic stress and cellular damage.

### SIRT2 is localized to cardiac mitochondria and interacts with CPT2

**Figure 5:**
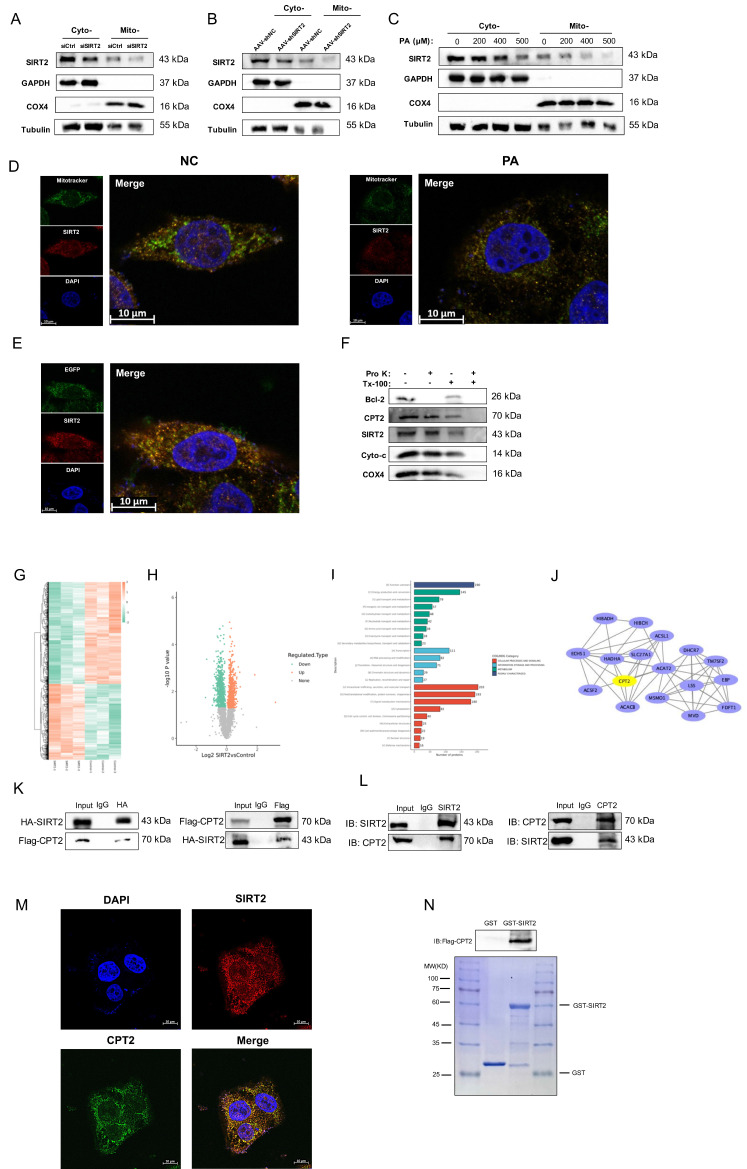
** Mitochondrial SIRT2 interacts with CPT2. (A)** siCtrl and siSIRT2 were used to interfere with the intracellular SIRT2 levels in NRCMs. Cytoplasmic and mitochondrial fractions were isolated from cardiomyocytes. The protein expression of SIRT2. COX4, GAPDH, and Tubulin were used as controls. **(B)** Cytoplasmic and mitochondrial fractions were isolated from AAV-shNC and AAV-shSIRT2 mouse heart tissues, and the protein expression of SIRT2 was measured. COX4, GAPDH, and tubulin were used as controls. **(C)** NRCMs were treated with 0 μM, 200 μM, 400 μM, or 500 μM PA for 24 hours. Cytoplasmic and mitochondrial fractions were isolated from cardiomyocytes. The protein expression of SIRT2. COX4, GAPDH, and tubulin were used as controls. **(D)** NRCMs were stained with MitoTracker, SIRT2 and DAPI, and representative IF images are shown. Scale bar: 10 μm. **(E)** NRCMs carrying the mitochondria-targeting plasmid pEGFP-MTS were stained with SIRT2 and DAPI, and representative IF images are shown. Scale bar: 10 μm. **(F)** Mitochondria (100 μg per treatment) from NRCMs were suspended in buffer with or without Triton X-100 (Tx-100) and proteinase K (Pro K). The samples were analyzed via western blotting. Bcl-2 (the outer mitochondrial membrane), CPT2 (the inner mitochondrial membrane), cytochrome C (the intermembrane space), and COX4 (the matrix) were used as markers. **(G)** Heatmap of representative differentially expressed genes in SIRT2-overexpressing AC16 cells. **(H)** Volcano plot of differentially expressed genes. Downregulation and upregulation are shown as green and red dots, respectively. **(I)** COG/KOG Category analysis of differentially expressed genes. **(J)** PPI network of lipid metabolism-related genes. **(K)** Co-IP experiments in HEK293T cells transfected with HA-SIRT2 and Flag-CPT2 using antibodies against HA or Flag, respectively, and normal IgG as a control. **(L)** Lysates from AC16 cells were extracted for co-IP with anti-SIRT2 or anti-CPT2 antibodies. **(M)** Representative images of endogenous immunofluorescence staining of AC16 cells costained with a SIRT2 antibody (red) and a CPT2 antibody (green). Nuclei were stained with DAPI (blue). Yellow indicates SIRT2 colocalization with CPT2 in AC16 cells. Scale bar, 50 μm. **(N)** *In vitro* GST pull-down assay between GST-SIRT2 and Flag-CPT2. Coomassie Brilliant Blue staining.

SIRT2 has been reported to be located in mitochondria 12. We found that SIRT2 regulated FA metabolism in the pathogenesis of DCM, so we speculate that SIRT2 not only is a cytosolic protein but also localizes to the mitochondria in cardiomyocytes. To test this hypothesis, we isolated mitochondrial lysates from NRCMs and DCM mice. The results showed that SIRT2 could be easily detected in mitochondrial lysates from NRCMs and heart tissues (Fig. 5A, B). Moreover, we extracted cytosolic and mitochondrial fractions from PA-treated NRCMs and detected that SIRT2 levels were reduced in mitochondria (Fig. 5C). SIRT2 mitochondrial localization was confirmed by costaining SIRT2 with MitoTracker in NRCMs and AC16 cells (Fig. 5D, Supplementary Fig. 11A).

In addition, the mitochondria-targeting plasmid pEGFP-mitochondrial targeting sequence (MTS) also overlapped with SIRT2 in NRCMs (Fig. 5E). To further confirm the precise location of SIRT2 in mitochondria, we isolated mitochondria and subjected them to a selective protein digestion assay (Fig. 5F, Supplementary Fig. 11B). The data showed that SIRT2 did not further decrease after proteinase K treatment, suggesting that SIRT2 is located in the inner membrane, matrix or intermembrane of mitochondria in cardiomyocytes. Accordingly, we claimed that SIRT2 was located in myocardial mitochondria. To examine the role of mitochondrial SIRT2 (mtSIRT2) in cardiac function, we constructed a SIRT2 variant with an MTS to ensure its localization to the mitochondria. Then, we transfected SIRT2-MTS into SIRT2 knockout cell line. Immunoblotting confirmed the expression of SIRT2 in the mitochondria (Supplementary Fig. 12A). We observed that mtSIRT2 could restored the impairment of PA-treated SIRT2 deletion cardiomyocytes, including cell apoptosis, inflammation, hypertrophy, fibrosis, and FAO (Supplementary Fig. 12B-D). These findings suggest mtSIRT2 plays an important role in cardiac protection.

Next, to identify the molecular mechanism underlying the positive effects of SIRT2 on DCM, we collected cells from the control group and the group stably overexpressing SIRT2 and performed TMT-labeling proteomics analysis to assess the underlying protein conditions. We identified 821 upregulated proteins and 1101 downregulated proteins (Fig. 5G, H). In the COG functional classification, the differentially expressed proteins were enriched in biological processes, including “energy production and conversion” and “lipid transport and metabolism” categories (Fig. 5I). We subsequently utilized Cytoscape software to analyze the protein interaction network of lipid transport and metabolism proteins and further used the network analysis plugin MCODE to calculate key proteins in the protein network (Fig. 5J). Among the targeted proteins, CPT2, a mitochondrial membrane protein, is an important regulator of FAO 33. Thus, it was hypothesized that SIRT2 might interact with CPT2. A coimmunoprecipitation (co-IP) assay confirmed that both exogenous (Fig. 5K) and endogenous (Fig. 5L) SIRT2 and CPT2 were precipitated. Furthermore, the colocalization of the SIRT2 and CPT2 proteins was further confirmed by endogenous immunofluorescence analysis (Fig. 5M). Additionally, we explored any potential interactions between SIRT4, SIRT5, and CPT2, revealing no associations among them (Supplementary Fig. 13). Finally, a GST pull-down assay confirmed that CPT2 could directly bind to GST-SIRT2 (Fig. 5N). Thus, cardiac mitochondrial SIRT2 plays a critical role in the regulation of DCM by binding with CPT2.

### Cardiac mitochondrial SIRT2 deacetylates CPT2 and contributes to its degradation

**Figure 6:**
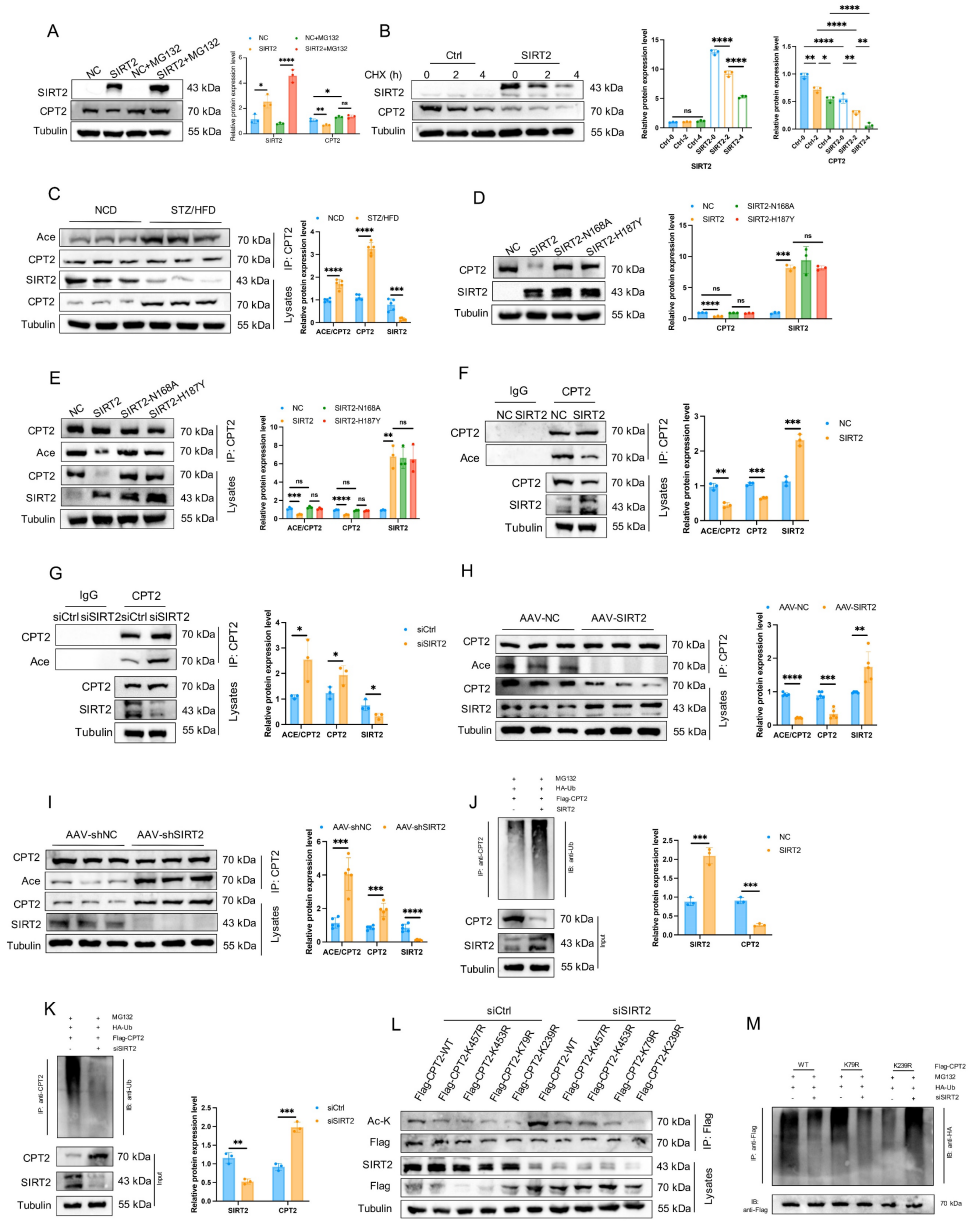
** Cardiac mitochondrial SIRT2 deacetylates CPT2 and contributes to its degradation. (A)** Protein levels of CPT2 with or without SIRT2 overexpression after treatment with the protease inhibitor MG132 (20 μM) for 10 hours (n=3). **(B)** Stability time course of CPT2 with or without SIRT2 overexpression after treatment with 100 μg/mL CHX for the indicated times (n=3). **(C)** Co-IP assay of cardiomyocytes from NCD or STZ/HFD mice, using an anti-CPT2 antibody for IP and anti-acetyl-lysine antibodies for immunoblotting (n=5). **(D)** SIRT2 and CPT2 protein expression levels in AC16 cells expressing empty vector (NC), SIRT2, or mutant plasmids (SIRT2-N168A, SIRT2-H187Y) (n=3). **(E)** Co-IP experiments of AC16 cells expressing NC or SIRT2 or mutant plasmids (SIRT2-N168A, SIRT2-H187Y), using an anti-CPT2 antibody for IP and anti-acetyl-lysine antibodies for immunoblotting (n=3). **(F)** Co-IP experiments of AC16 cells overexpressing NC or SIRT2 plasmids, using an anti-CPT2 antibody for IP and anti-acetyl-lysine antibodies for immunoblotting (n=3). **(G)** Co-IP experiments of AC16 cells transfected with siCtrl or siSIRT2 using an anti-CPT2 antibody for IP and anti-acetyl-lysine antibodies for immunoblotting (n=3). **(H)** Co-IP assay of cardiomyocytes from AAV-NC or AAV-SIRT2 mice using an anti-CPT2 antibody for IP and anti-acetyl-lysine antibodies for immunoblotting (n=5). **(I)** Co-IP assay of cardiomyocytes from AAV-shNC- or AAV-shSIRT2-treated mice; an anti-CPT2 antibody was used for IP, and an anti-acetyl-lysine antibody was used for immunoblotting (n=5). **(J-K)** AC16 cells transfected with Flag-CPT2 and the indicated plasmids or siRNAs were treated with 10 μM MG132 for 6 h. The level of CPT2 ubiquitination was probed with an anti-Ub antibody (n=3). **(L)** siCtrl or siSIRT2 was transfected into HEK293T cells with either CPT2-WT or different mutant plasmids, including K457R, K453R, K79R and K239R. Lysates were immunoprecipitated with an anti-Flag antibody and blotted with an anti-acetyl-lysine antibody (n=3). **(M)** Ubiquitination levels of IP-purified Flag-CPT2 and its mutants in HEK293T cells with SIRT2 knockdown (n=3). The data are presented as the mean ± SD. *p<0.05; **p<0.01; ***p<0.001; ****p<0.0001; ns indicates no significance. Statistical analysis was performed via one-way ANOVA and unpaired Student's t test.

Acetylation and deacetylation play central roles in regulating metabolic enzyme activity and protein stability 34. We thus asked whether SIRT2 affects the acetylation of CPT2. Western blotting analysis revealed that SIRT2 overexpression reduced CPT2 protein levels in mouse hearts, whereas SIRT2 deficiency increased CPT2 protein levels (Supplementary Fig. 14A, B). However, qRT-PCR indicated that SIRT2 did not affect CPT2 transcription (Supplementary Fig. 14C, D). Consistently, plasmid-mediated SIRT2 overexpression *in vitro* reduced CPT2 protein levels, whereas siRNA-mediated SIRT2 deficiency increased CPT2 protein levels without affecting CPT2 transcription (Supplementary Fig. 14E-H). These results suggest that SIRT2 can interact with CPT2 to affect its protein stability. To further confirm this, we detected the protein levels of CPT2 in AC16 cells with or without SIRT2 overexpression after treatment with the proteasome inhibitor MG132. The results indicated that SIRT2 overexpression promoted CPT2 protein destabilization (Fig. 6A). Then, we tested the half-life of CPT2 after treatment with CHX. The results showed that SIRT2 overexpression reduced the half-life of the CPT2 protein (Fig. 6B). Overall, these findings suggested that the interaction between SIRT2 and CPT2 was responsible for its protein stability.

We hypothesized that SIRT2 might regulate CPT2 protein stability via deacetylation. Indeed, overall acetylation levels in cardiomyocytes and the myocardium increased in both PA-treated cells and STZ/HFD-induced mice (Supplementary Fig. 15). Examination of CPT2 acetylation in heart tissues from STZ/HFD-fed mice and PA-treated AC16 cells revealed that, compared with NCD-fed mice, STZ/HFD-fed mice presented significantly increased CPT2 levels and acetylation in the heart (Fig. 6C). Similar results were observed in PA-treated AC16 cells (Supplementary Fig. 16A, B). To determine whether SIRT2 regulates the function of CPT2 through its deacetylase activity, we conducted an* in vitro* SIRT2 deacetylation assay. AC16 cells were transfected with either wild-type SIRT2 or catalytic mutant (N168A and H187Y) plasmids. The overexpression of WT-SIRT2 decreased CPT2 expression, whereas the catalytically inactive mutants had no effect (Fig. 6D). Additionally, SIRT2 overexpression reduced CPT2 acetylation, but the mutants did not (Fig. 6E). *In vivo* and *in vitro* deacetylation assays in AC16 cells revealed that SIRT2 overexpression decreased CPT2 acetylation, whereas SIRT2 knockdown increased it (Fig. 6F-I). Similar results were obtained via IP of acetyl-lysine followed by western blotting for CPT2 (Supplementary Fig. 16C-G), confirming that SIRT2 deacetylates CPT2. Importantly, SIRT2 overexpression significantly increased CPT2 ubiquitination, whereas SIRT2 knockdown significantly decreased it (Fig. 6J, K), indicating that SIRT2-mediated deacetylation increases CPT2 ubiquitination.

To map the deacetylation site of CPT2 by SIRT2, we analyzed 4 acetylation sites. The K457, K453, and K79 sites were predicted by existing scholarly literature, and the K239 sites were confirmed by mass spectrometry (Supplementary Fig. 16H). We mutated these sites to arginines (R, nonacetylatable) via site-directed mutagenesis. The cells were transfected with plasmids expressing CPT2-WT or different CPT2 mutants, along with siCtrl or siSIRT2. CPT2-K457R, CPT2-K453R, and CPT2-K79R acetylation levels increased significantly in SIRT2-knockdown cells, but there was no difference in CPT2-K239R acetylation levels (Fig. 6L), indicating that K239 is an acetylation site. To determine whether the acetylation mutation affects CPT2 stability, HEK293T cells transfected with CPT2-WT or the CPT2-K239R mutant were treated with CHX. Compared with CPT2-WT, the CPT2-K239R mutant presented a shorter half-life, suggesting that K239 acetylation is related to CPT2 protein stability (Supplementary Fig. 16I). Additionally, the K239R mutation reversed the reduction in ubiquitination levels caused by SIRT2 knockdown (Fig. 6M). These findings suggest that SIRT2-mediated deacetylation at K239 plays a crucial role in regulating CPT2 protein stability.

### CPT2 mediates the effects of cardiac mitochondrial SIRT2 in DCM

**Figure 7:**
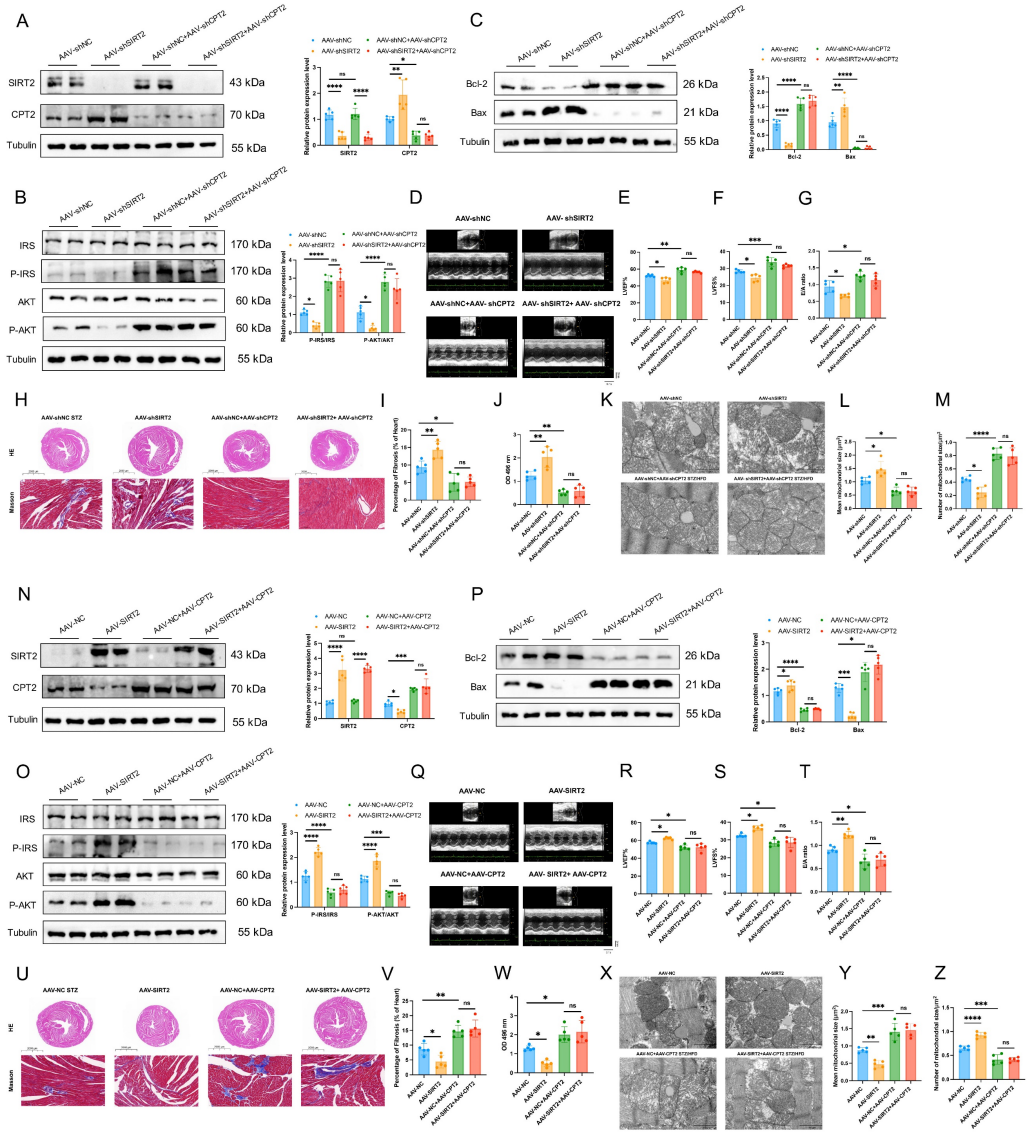
** Simultaneous deficiency/overexpression of SIRT2 and CPT2 improves/inhibits insulin resistance, cell apoptosis, cardiac dysfunction, and mitochondrial dysfunction in DCM male mice. (A, N)** C57BL/6J male mice were fed with STZ/HFD for 20 weeks after being injected with 1×10^11^ pfu AAV-shNC (n=5), AAV-shSIRT2 (n=5), AAV-shNC+AAV-shSIRT2 (n=5), AAV-shSIRT2+AAV-shCPT2 (n=5), AAV-NC (n=5), AAV-SIRT2 (n=5), AAV-NC+AAV-SIRT2 (n=5), or AAV-SIRT2+AAV-CPT2 (n=5) through the tail vein. The protein expression of SIRT2 and CPT2 in mouse heart tissue after virus injection and induction with STZ/HFD. **(B, O)** Protein expression of insulin receptor signaling in mouse heart tissue. **(C, P)** Protein expression of the apoptosis gene Bax and the anti-apoptotic gene Bcl2 in mouse heart tissue. **(D, Q)** Representative images of echocardiograms. **(E, R)** The percentage of LVEF was determined. **(F, S)** The percentage of LVFS was determined. **(G, T)** E/A ratio. **(H, U)** Representative images of heart sections stained with HE (top) and Masson (bottom) are shown. Scale bars, 2000 μm (HE), and 100 μm (Masson).** (I, V)** Quantification of the Masson staining area in heart sections is shown. **(J, W)** Representative FAO levels in heart tissues. **(K, X)** Representative electron microscopy images of heart sections. Scale bar, 1 µm.** (L, Y)** Mean mitochondrial size (expressed as μm^2^) in heart sections. **(M, Z)** Mitochondrial numbers (expressed per μm^2^) in heart sections. The data are presented as the mean ± SD. *p<0.05; **p<0.01; ***p<0.001; ****p<0.0001; ns indicates no significance. Statistical analysis was performed via one-way ANOVA.

We further asked whether CPT2 mediated the effects of SIRT2 on DCM. To this end, we first investigated whether CPT2 knockdown reversed the adverse effects of SIRT2 knockdown in an *in vivo* model. AAV-shNC, AAV-shSIRT2, AAV-shNC+AAV-shCPT2, and AAV-shSIRT2+AAV-shCPT2 mice were established (Fig. 7A and Supplementary Fig. 17A) and subjected to STZ/HFD challenge. Our results showed that a reduction in CPT2 offset the increase in body weight and FBG induced by SIRT2 knockout (Supplementary Fig. 18A, B). Moreover, both CPT2-knockdown groups presented similar improvements in glucose and insulin tolerance (Supplementary Fig. 18C, D). CPT2 silencing attenuated the damage to insulin signaling, cell apoptosis, and cardiac dysfunction induced by SIRT2 deficiency (Fig. 7B-I, Supplementary Fig. 18E, Supplementary Fig. 19A). Moreover, we also found that knockdown of SIRT2 increased the levels of heart FFAs and FAO but not in CPT2-deficient mice (Fig. 7J, Supplementary Fig. 18F). Considering that cardiac lipotoxicity is closely correlated with mitochondrial dysfunction, we also examined mitochondrial morphology via transmission electron microscopy (TEM). The data revealed that the mitochondria exhibited obvious irregularity, swelling, and disarrayed cristae in the AAV-shSIRT2 mice. However, the mitochondria in the AAV-shNC+AAV-shCPT2 and AAV-shSIRT2+AAV-shCPT2 groups were healthy with orderly arranged cristae, the mean mitochondrial size was decreased, and the number of mitochondria per μm^2^ was increased (Fig. 7K-M). We subsequently generated AAV-NC, AAV-SIRT2, AAV-NC+AAV-CPT2 and AAV-SIRT2+AAV-CPT2 STZ/HFD-fed mice (Fig. 7N and Supplementary Fig. 17B). In the context of CPT2 overexpression, SIRT2 overexpression did not improve body weight, FBG, glucose tolerance, insulin tolerance, insulin signaling, cell apoptosis, cardiac dysfunction, heart FFAs, FAO, or mitochondrial dysfunction (Fig. 7O-Z, Supplementary Fig. 18G-L, Supplementary Fig. 19B). Similarly, we used siRNA to silence the CPT2 and SIRT2 genes in AC16 human cardiomyocytes (Supplementary Fig. 20A). CPT2 knockdown counteracted the SIRT2 knockdown-mediated exacerbation of cell apoptosis, hypertrophy, and fibrosis (Supplementary Fig. 20B-D), and siCPT2 downregulated LD accumulation (Supplementary Fig. 20E, F) and further decreased ROS (Supplementary Fig. 20G, H). Moreover, cells were treated with HA-SIRT2 and Flag-CPT2 plasmids to increase the expression of the CPT2 and SIRT2 genes (Supplementary Fig. 20I). CPT2 overexpression aggravated cell apoptosis, hypertrophy, and fibrosis (Supplementary Fig. 20J-L), as did LD accumulation and ROS (Supplementary Fig. 20M-P).

Meanwhile, to further investigate the functional significance of the CPT2-K239 acetylation site in DCM, we combined the CPT2-K239R mutant plasmid with the SIRT2 plasmid to validate that the protective effect of SIRT2 on cardiomyocytes is dependent on CPT2. Overexpression of mutant CPT2 significantly improved PA-induced cell apoptosis, inflammation, hypertrophy and fibrosis, and suppressed FAO. Transfection of CPT2-K239R and simultaneous overexpression of SIRT2 in cardiomyocytes had the same effect (Supplementary Fig. 21). These data indicated that SIRT2 inhibited cell apoptosis, inflammation, cardiac dysfunction, and FAO partially through Lys239 deacetylation of CPT2. Collectively, these data indicate that SIRT2 inhibits insulin resistance, cell apoptosis, cardiac dysfunction, and metabolic profile impairments partially through the Lys239 deacetylation of CPT2.

## Discussion

Our study revealed a previously unrecognized role for cardiac mitochondrial SIRT2 in regulating DCM. SIRT2 protects against cardiac dysfunction in DCM by regulating mitochondrial FAO. For the first time, we show that SIRT2 is localized in the mitochondria of cardiomyocytes, and restoring cardiac-specific SIRT2 expression is sufficient to alleviate cardiac pathophysiological alterations and improve cardiomyocyte function. Furthermore, our data indicate that SIRT2 modulates mitochondrial fatty acid metabolism in the heart, with CPT2, a mitochondrial FAO enzyme, serving as a deacetylated target mediating the cardioprotective effects of SIRT2. Mechanistically, SIRT2 deacetylates CPT2 at K239, leading to a decrease in its protein stability. Collectively, our results demonstrate that cardiac mitochondrial SIRT2 could serve as a potential therapeutic target for diabetic cardiomyopathy and related metabolic disorders.

The sirtuin family contains seven mammalian members (SIRT1-SIRT7), which are homologous to the yeast silent information regulator 2 (Sir2) gene 17, 35. Data from the literature indicate that all sirtuins regulate one another in a complex network, coordinating cardiac physiology and preserving proper function. Jin *et al.* demonstrated that the FGF21-SIRT3 axis mediated the protective effects of exercise against DCM by preserving mitochondrial integrity 23. Wei *et al.* found that SIRT5-mediated lysine demalonylation of GSTP1 protected against oxidative stress and pyroptosis in DCM mice 28. Wu *et al.* reported that SIRT6 was a novel regulator of FA transport and diastolic dysfunction under diabetic conditions 31. Moreover, studies have shown that SIRT1 activation appears to be a promising tool for the protection of diabetic hearts. However, the role of SIRT2 in the heart has yet to be completely elucidated. SIRT2 is an NAD^+^-dependent deacetylase whose enzymatic activity is linked to the energy state of the cell. It is highly expressed in the brain and heart of humans. The activation of SIRT2 is believed to be beneficial for metabolic diseases, such as cancer, type II diabetes, and obesity 36, 37. In an *in vitro* study, insulin resistance due to diet-induced obesity was exacerbated by the knockout of the SIRT2 gene 38. Additionally, SIRT2 may modulate cell proliferation and apoptosis by regulating autophagy 35. Notably, conflicting evidence exists regarding the role of SIRT2 in cardiac hypertrophy. One study showed that SIRT2 deficiency could inactivate AMPK signaling, promoting cardiac hypertrophy 17. However, another study found that SIRT2 had detrimental effects on the heart and played a role in the cardiac response to injury and the progression of cardiac hypertrophy 39. Notably, our study aimed to elucidate the pivotal role of SIRT2 in the pathogenesis of diabetic cardiomyopathy, which is characterized by distinct alterations in cardiac energy metabolism. Retroviral expression of SIRT2 in adipocytes promotes lipolysis 14. SIRT2 plays a critical role in protecting cells from oxidative damage by maintaining NADPH homeostasis 40. Thus, these data suggested a protective role of SIRT2 in the development of metabolic dysfunction, and it is reasonable to investigate whether SIRT2 exerts a protective effect against DCM. Our detailed analysis revealed that SIRT2 expression was significantly decreased in the STZ/HFD challenge-induced DCM mouse model, indicating the potential role of SIRT2 in this disease. Moreover, using AAV-mediated delivery of cardiac SIRT2 into STZ/HFD-induced diabetic mice, we observed that SIRT2 overexpression significantly alleviated type II diabetes-related insulin resistance, cell apoptosis, and cardiac dysfunction. The inversion results were detected in the AAV-shSIRT2 STZ/HFD-fed mice. We also found that cardiac-specific overexpression or downregulation of SIRT2 could influence peripheral glucose and lipid metabolism, possibly through the role of SIRT2 in cardiac inflammation. IL-6 has been proposed to play a role in glucose homeostasis and metabolism, directly and indirectly through its action on skeletal muscle cells, adipocytes, hepatocytes, and pancreatic β-cells 41. These results provide* in vivo* evidence supporting the crucial role of cardiac SIRT2 in the pathogenesis of DCM.

DCM is commonly related to cardiac lipotoxicity, which likely leads to cardiac dysfunction 42. In the diabetic heart, the use of glucose is reduced, and the oxidation of FFAs is increased. Concurrently, superfluous FAs are converted to toxic intermediates or diverted to cellular-neutral lipid pools 43, 44. Accumulating evidence suggests that modulating cardiac energy metabolism by reducing fatty acid oxidation can improve cardiac function in DCM. Notably, our results indicated that SIRT2 decreased LD accumulation, FAO, and OCR, ultimately reducing ROS production and thereby preventing the development of DCM. Therefore, our findings revealed that SIRT2 participated in fatty acid metabolism. Mitochondria and fatty acids are tightly connected to multiple cellular processes. It has previously been shown that nonmitochondrial sirtuins, specifically SIRT1, can regulate mitochondrial metabolism 12. SIRT2 can be localized in cerebral mitochondria, but whether it can be localized in myocardial mitochondria and the relationship between SIRT2 and DCM are unclear. In this study, we showed that cytoplasmic SIRT2 also directed mitochondrial fatty acid metabolism and relocated to cardiac mitochondria. This localization of SIRT2 might allow it to deacetylate its mitochondrial targets, thereby regulating the activity of mitochondria, such as energy production, FAO, and ROS. Our results support the notion that cardiac mitochondrial SIRT2 has a protective effect on DCM by regulating cardiac fatty acid oxidation.

The carnitine palmitoyltransferase (CPT) system is a mitochondrial enzyme involved in FAO regulation 45. The CPT family is composed of two separate proteins: CPT1, which is located in the outer membrane, and CPT2, which is located in the inner membrane of the mitochondria 46. CPT2 is a ubiquitous enzyme involved in FAO regulation. Some researchers have shown that deficiency or dysfunction contributes to critical roles in lipid metabolic diseases, such as obesity, diabetes and NAFLD 46-48. Loss of muscle CPT2 results in a high degree of long-chain acylcarnitine accumulation, which has been shown to protect against diet-induced obesity and insulin resistance 45. Fujiwara *et al.* reported the downregulation of CPT2 and massive accumulation of acylcarnitine in the HCC tissue and serum of HFD-fed mice. CPT2 deficiency in HCC cells inhibits FA β-oxidation and suppresses the activation of JNK mediated by Src, which results in the avoidance of lipotoxicity 19. Therefore, CPT2 is essential for regulating FAO and plays a critical role in DCM development. As expected, in our study, cardiac mitochondrial SIRT2 directly interacted with CPT2. SIRT2 overexpression decreased CPT2 protein expression and promoted insulin resistance, cell apoptosis, and cardiac dysfunction both *in vivo* and *in vitro*. Our results also demonstrated that CPT2 overexpression blocked the cardioprotective effects of SIRT2. These results indicate that CPT2 is a downstream target of cardiac mitochondrial SIRT2, which modulates the development of DCM by decreasing CPT2 expression.

Lysine acetylation has recently emerged as an important posttranslational modification that can regulate the activity and function of several enzymes involved in fatty acid and glucose metabolism 49. Studies have shown that dietary interventions can stimulate sirtuins and deacetylate multiple mitochondrial proteins, indicating that acetylation is related to mitochondrial dynamics, such as metabolism, apoptosis, and the stress response. Liu *et al.* demonstrated that CPT2 K453/K457 were the sites of SIRT3-mediated CPT2 activation, which was essential for CPT2 dimerization in LPA-induced mitochondrial metabolic enhancement 47. Fan *et al.* identified an acetylation site in the CPT2 protein and reported that K79 acetylation was the primary cause of the blockade of FAO and the accumulation of long-chain acylcarnitines (LCACs) 20. These results suggested that CPT2 could be acetylated. In our study, mass spectrometry was performed to examine the acetylation site of CPT2, and the K239 residue was confirmed. Combining the acetylation sites reported in previous studies and identified by mass spectrometry, we compared the changes in these acetylation sites of CPT2 under si-Ctrl or si-SIRT2 conditions. We demonstrated that SIRT2 binds to and deacetylates CPT2 at the K239 residue, thereby decreasing CPT2 protein levels through its degradation. Moreover, unlike the regulation of transcriptional activity by deacetylation, the current data reveal, for the first time, that the deacetylation switch may regulate CPT2 protein ubiquitination and stability. Thus, lysine deacetylation at K239 is a posttranslational modification that regulates CPT2 protein stability, contributing to the protective role of cardiac mitochondrial SIRT2 in DCM.

In summary, our study reveals the critical role of cardiac mitochondrial SIRT2 as a key metabolic regulator in diabetic hearts. The upregulation of SIRT2 expression improves insulin resistance, cell apoptosis, and cardiac dysfunction, mainly by deacetylating and disrupting CPT2 expression. Our work strongly suggests that specifically targeting the cardiac mitochondrial SIRT2/CPT2 regulatory axis may serve as a determinant of DCM and a promising therapeutic target for DCM.

## Supplementary Material

Supplementary figures and tables.

## Abbreviations

DCM: diabetic cardiomyopathy
DM: diabetes mellitus
T2D: type II diabetes mellitus
SIRT2: sirtuin 2
STZ/HFD: streptozotocin/high-fat diet
NCD: normal chow diet
IR: insulin resistance
CPT2: carnitine palmitoyltransferase 2
ROS: reactive oxygen species
HF: heart failure
FAO: fatty acid oxidation
PA: palmitic acid
FBG: fasting blood glucose
IPGTT: intraperitoneal glucose tolerance test
IPITT: intraperitoneal insulin tolerance test
NRCMs: neonatal rat cardiomyocytes
HR: heart rate
LVEF: left ventricular ejection fraction
LVFS: left ventricular fraction shortening
co-IP: coimmunoprecipitation
LD: lipid droplet
OCRs: oxygen consumption rates
HW/TL: heart weight-to-tibia length ratio
ANP: atrial natriuretic polypeptide
BNP: brain natriuretic peptide
COL1A1: collagen 1a1
COL3A1: collagen 3a1
MTS: mitochondrial targeting sequence
mtSIRT2: mitochondrial SIRT2
