# Supplementary material for: Mitochondrial SIRT2-mediated CPT2 deacetylation prevents diabetic cardiomyopathy by impeding cardiac fatty acid oxidation
Source: Int J Biol Sci. 2025 Jan 1;21(2):725–44. doi: 10.7150/ijbs.102834 (PMC11705638; doi:10.7150/ijbs.102834)
Supplement: Supplementary file 1 — Supplementary figures and tables. [file ijbsv21p0725s1.pdf]

Mitochondrial SIRT2-mediated CPT2 deacetylation prevents diabetic cardiomyopathy by impeding cardiac fatty acid oxidation

Yaoyao Guo[1,2,3], Ziyin Zhang[1,2,3], Zheng Wen[4,5], Xiaonan Kang[1,2,3], Dan Wang[1,2,3], Lu Zhang[1,2,3], Mengke Cheng[1,2,3], Gang Yuan[1,2,3*], and Huihui Ren[1,2,3*]

1. Division of Endocrinology, Department of Internal Medicine, Tongji Hospital, Tongji Medical College, Huazhong University of Science and Technology, Wuhan, China.

2. Hubei Clinical Medical Research Center for Endocrinology and Metabolic Diseases, Hubei, China.

3. Branch of National Clinical Research Center for Metabolic Diseases, Hubei, China.

4. Division of Cardiology, Department of Internal Medicine, Tongji Hospital, Tongji Medical College, Huazhong University of Science and Technology, Wuhan, China.

5. Hubei Key Laboratory of Genetics and Molecular Mechanisms of Cardiological Disorders, Hubei, China.

[*]Corresponding author: Gang Yuan and Huihui Ren. Division of Endocrinology, Department of Internal Medicine, Tongji Hospital, Tongji Medical College, Huazhong University of Science and Technology, Wuhan, China. Tel and Fax: +86-27-8366-2883. Email: gangyuan@tjh.tjmu.edu.cn or huizi9258@163.com.

This file includes:

Figures S1-S21 and Legends

Table S1. Primers for plasmids construction.

Table S2. The antibodies were used in this study.

Table S3. Sequence of primers for PCR.

Figure S1

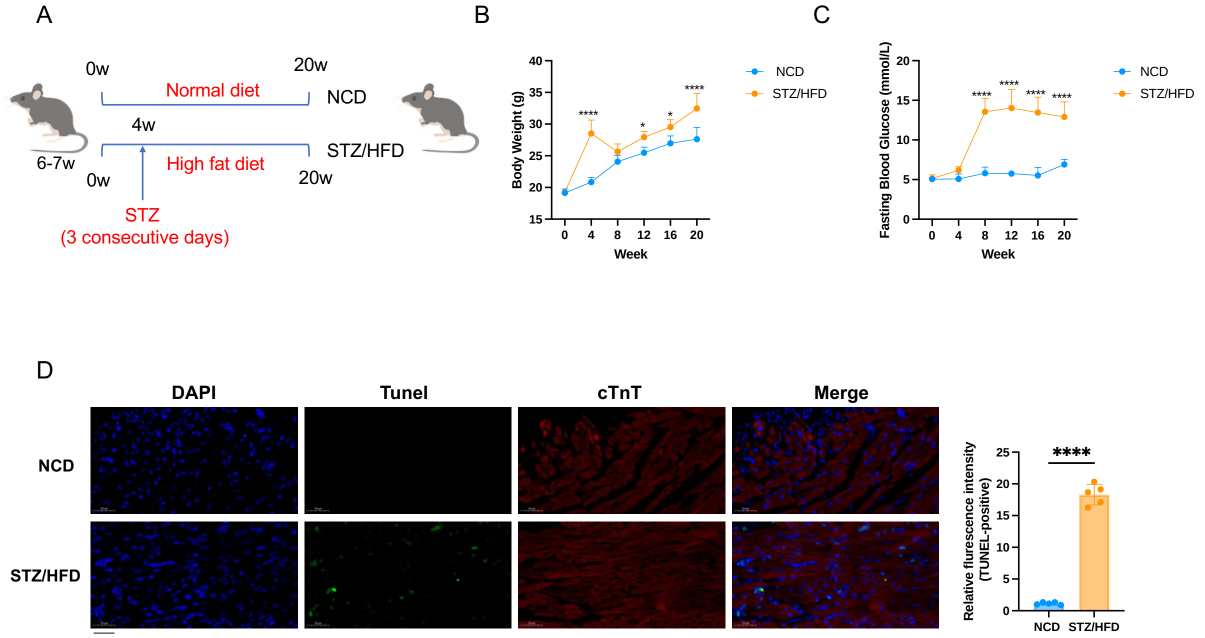

Supplementary Figure 1. Characterization of an experimental male mice model of DCM. (A) Schematic of the STZ/HFD-induced DCM model. **(B-C)** Body weight gain and fasting blood glucose of the mice over time after normal diet or STZ/HFD (n=5). **(D)** TUNEL assay in mouse heart tissues co-staining with cTnT (n=5). Scale bar, 50 µm. Data were presented as the mean ± SD. *p<0.05; ****p<0.0001. Statistical analysis was performed by unpaired Student's t test and two-way ANOVA.

Figure S2

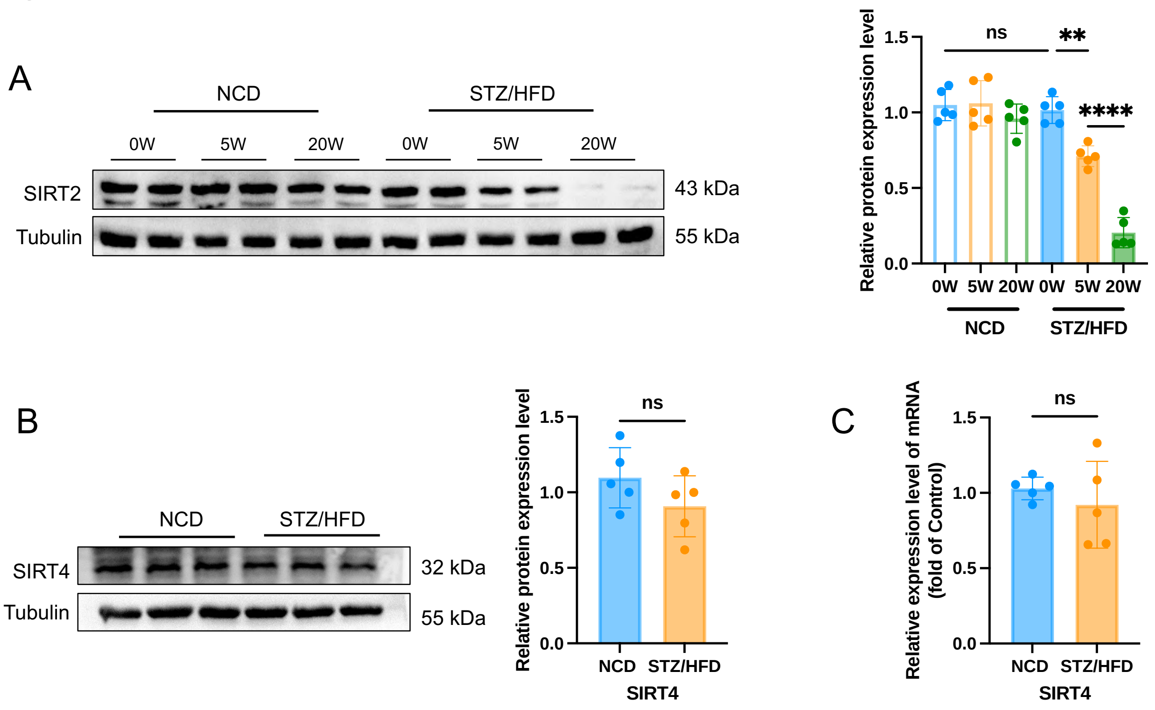

Supplementary Figure 2. The expression levels of SIRT2 and SIRT4 in the heart tissues of DCM. (A) The protein expression of SIRT2 was detected in the cardiac tissue of NCD and STZ/HFD-fed mice at different time points. **(B-C)** The protein and mRNA expression levels of SIRT4 in the cardiac tissues. (n=5). Data were presented as the mean ± SD. **p<0.01; ****p<0.0001; ns indicated no significance. Statistical analysis was performed by unpaired Student's t test and one-way ANOVA.

Figure S3

A

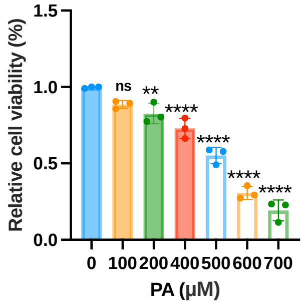

Supplementary Figure 3. (A) The viability of PA-treated cardiomyocytes was examined using CCK-8 assays (n=3). Data were presented as the mean ± SD. **p<0.01; ****p<0.001; ns indicated no significance. Statistical analysis was performed by one-way ANOVA.

Figure S4

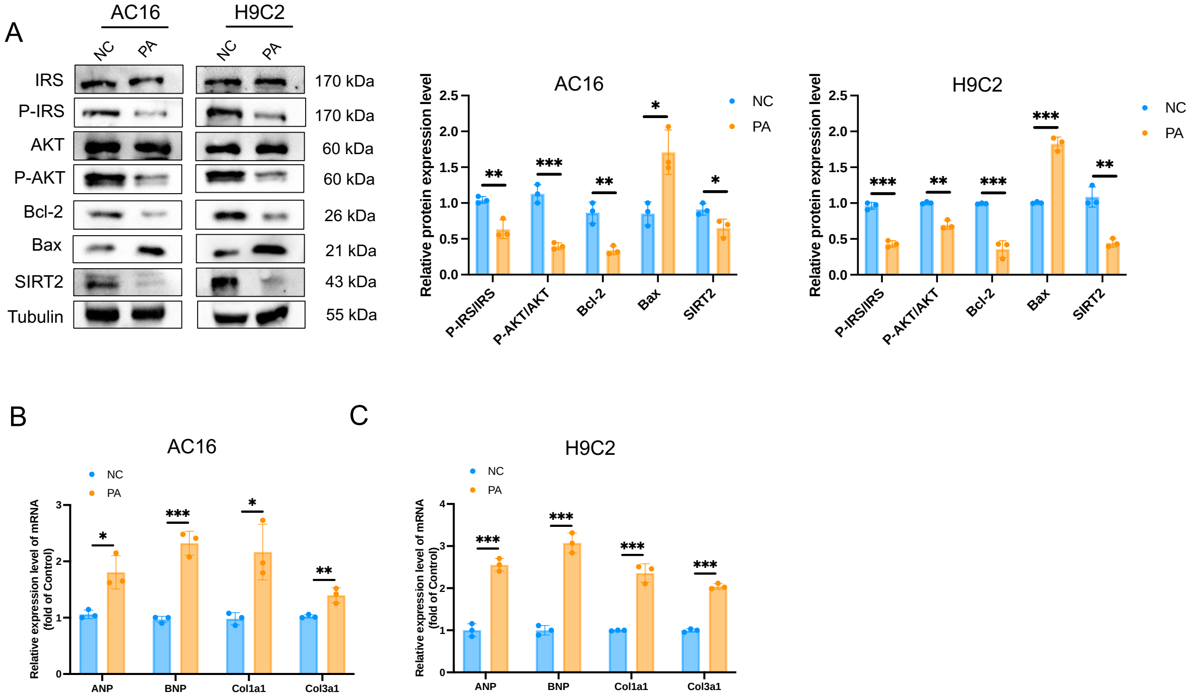

Supplementary Figure 4. The levels of insulin resistance, cell apoptosis, hypertrophy and fibrosis in PA-induced cardiomyocytes. (A) AC16 and H9c2 cells were treated with 0.5 mM PA for 24 h. Protein levels of insulin signaling pathway was measured after insulin (100 nmol/L) used for 10 min. Protein levels of apoptosis gene Bax and anti-apoptosis gene Bcl2 in cardiomyocytes were measured. **(B-C)** Hypertrophic genes and fibrotic genes mRNA expression in AC16 cells (B) and H9c2 cells (C) were tested by qRT-PCR. (n=3). Data were presented as the mean ± SD. *p<0.05; **p<0.01; ***p<0.001. Statistical analysis was performed by unpaired Student's t test.

Figure S5

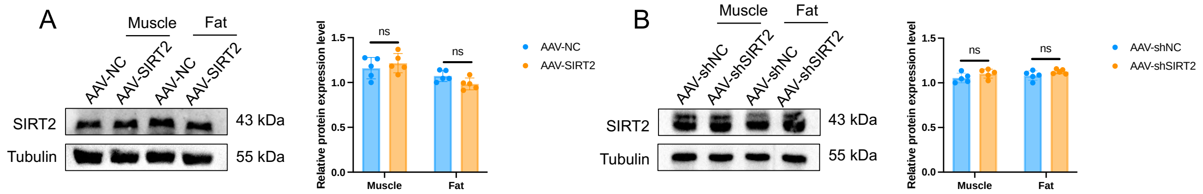

Supplementary Figure 5. Cardiac-specific SIRT2 overexpression/deficiency male mice are established by AAV. (A-B) The protein expression of SIRT2 in the muscle and fat. (n=5). Data were presented as the mean ± SD. ns indicated no significance. Statistical analysis was performed unpaired Student's t test.

Figure S6

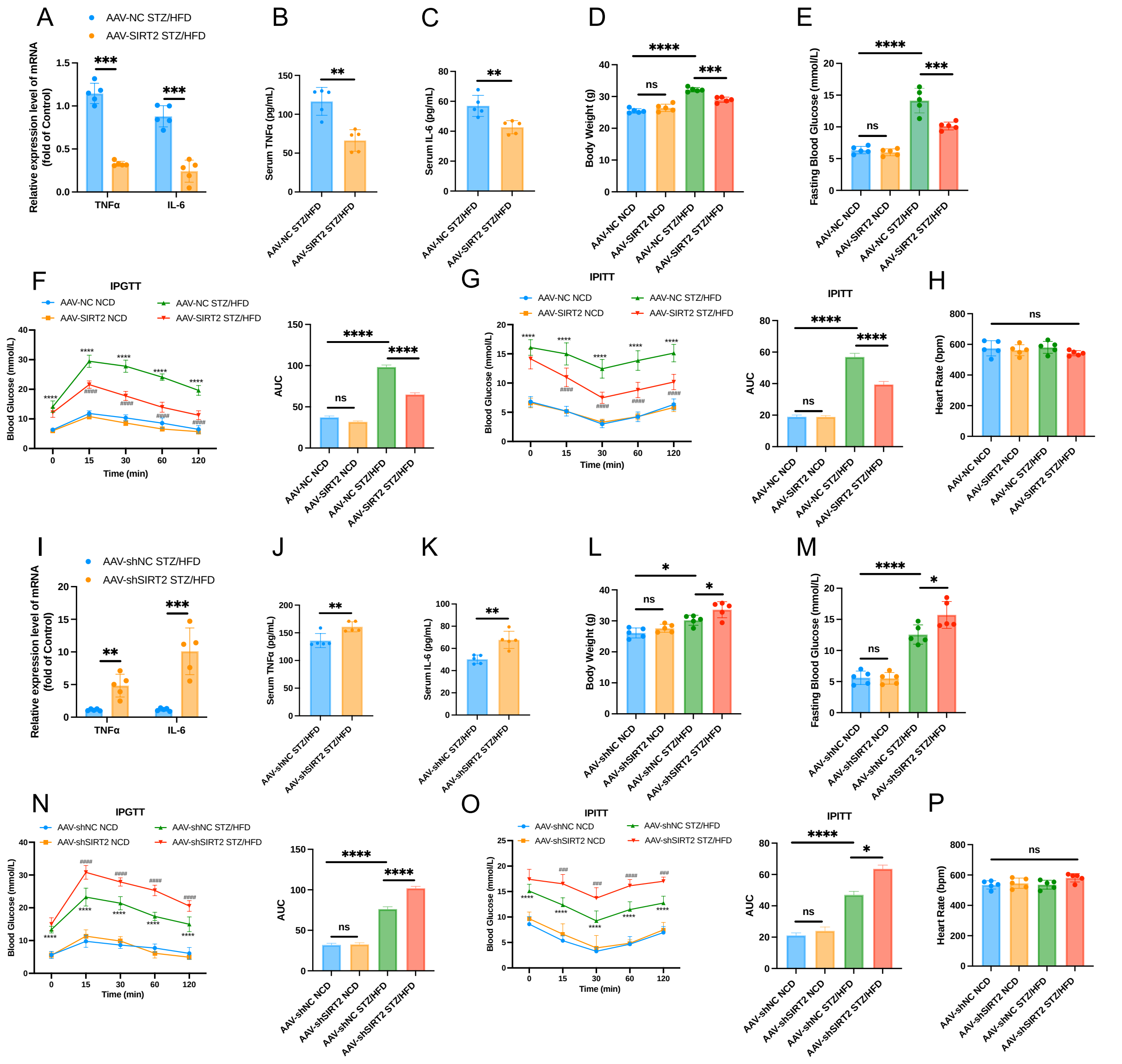

Supplementary Figure 6. Cardiac-specific SIRT2 overexpression/deficiency male mice improves/aggravates cardiac inflammatory and peripheral glycolipid metabolism. (A) Expression of inflammatory genes in heart tissues of AAV-NC STZ/HFD and AAV-SIRT2 STZ/HFD mice at mRNA level was examined, gene expression was normalized using β-Actin gene as standard. **(B-C)** The serum levels of TNF-α and IL-6 in mice were detected (pg/mL) by ELISA kits. **(D-E)** Representative body weight and fasting blood glucose in mice. **(F-G)** IPGTT/IPITT was performed after feeding for 20 weeks. Blood glucose levels were examined at the indicated times post-glucose/insulin injection. The area under the curve was calculated. # represented AAV-SIRT2 STZ/HFD and AAV-NC STZ/HFD, and * represented AAV-NC STZ/HFD and AAV-NC NCD. **(H)** Representative the heart rate in mice. **(I)** Expression of inflammatory genes in heart tissues of AAV-shNC STZ/HFD and AAV-shSIRT2 STZ/HFD mice at mRNA level was examined, gene expression was normalized using β-Actin gene as standard. **(J-K)** The serum levels of TNF-α and IL-6 in mice were detected (pg/mL) by ELISA kits. **(L-M)** Representative body weight and fasting blood glucose in mice. **(N-O)** IPGTT/IPITT was performed in 20-week-old mice. Blood glucose levels were examined at the

indicated times post-glucose/insulin injection. The area under the curve was calculated. # represented AAV-shSIRT2 STZ/HFD and AAV-shNC STZ/HFD, and * represented AAV-shNC STZ/HFD and AAV-shNC NCD. **(P)** Representative the heart rate in mice. (n=5). Data were presented as the mean ± SD. *p<0.05; **p<0.01; ***p<0.001; ****p<0.0001; ###p<0.001; ####p<0.0001; ns indicated no significance. Statistical analysis was performed by one-way ANOVA, two-way ANOVA, and unpaired Student's t test.

Figure S7

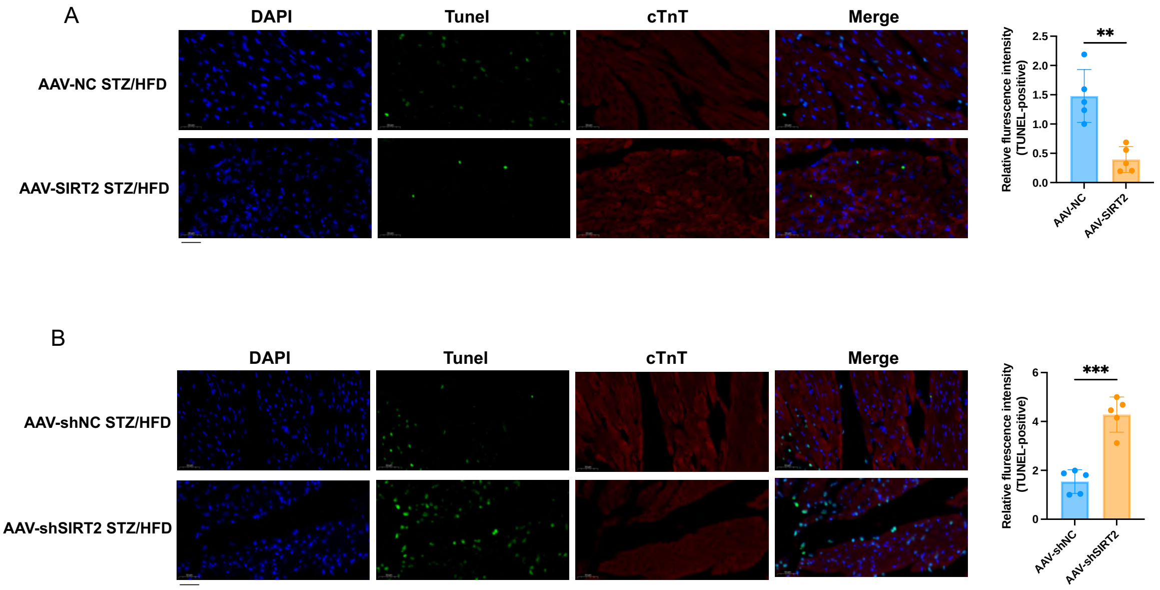

Supplementary Figure 7. Cardiac-specific SIRT2 overexpression/deficiency male mice improves/aggravates cell apoptosis in DCM male mice. (A-B) TUNEL assay in mouse heart tissues co-staining with cTnT. Scale bar, 50 μm. (n=5). Data were presented as the mean ± SD. $^{**}p<0.01$; $^{***}p<0.001$. Statistical analysis was performed by unpaired Student's t test.

Figure S8

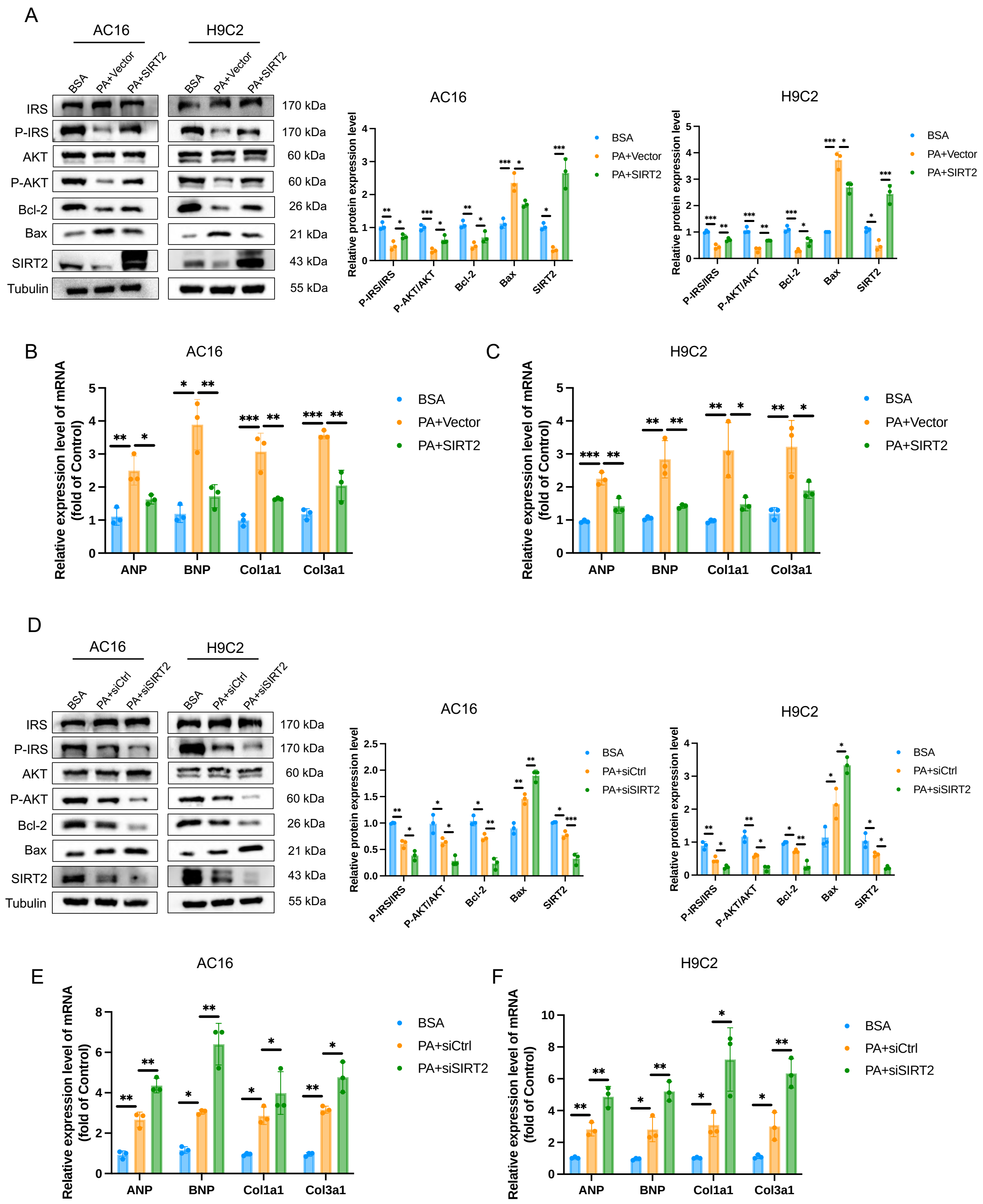

Supplementary Figure 8. Overexpression/deficiency of SIRT2 improves/impaires PA-induced insulin resistance, cell apoptosis, hypertrophy, and fibrosis. Vector and SIRT2 plasmids were used to increase intracellular SIRT2 levels in AC16 and H9c2 cells, followed by PA treated for 24 h. siCtrl and siSIRT2 were used to interfere with intracellular SIRT2 levels in AC16 and H9c2 cells, followed by PA treated for 24 h. BSA acted as control. **(A, D)** Protein levels of insulin signaling pathway was measured after insulin (100 nmol/L) used for 10 min. Protein levels of apoptosis protein Bax and anti-apoptosis Bcl2 in cardiomyocytes were

measured. **(B, E)** Hypertrophic genes and fibrotic genes mRNA expression in AC16 cells were tested by qRT-PCR. **(C, F)** Hypertrophic genes and fibrotic genes mRNA expression in H9c2 cells were tested by qRT-PCR. (n=3). Data were presented as the mean ± SD. *$p<0.05$; **$p<0.01$; ***$p<0.001$. Statistical analysis was performed by one-way ANOVA.

Figure S9

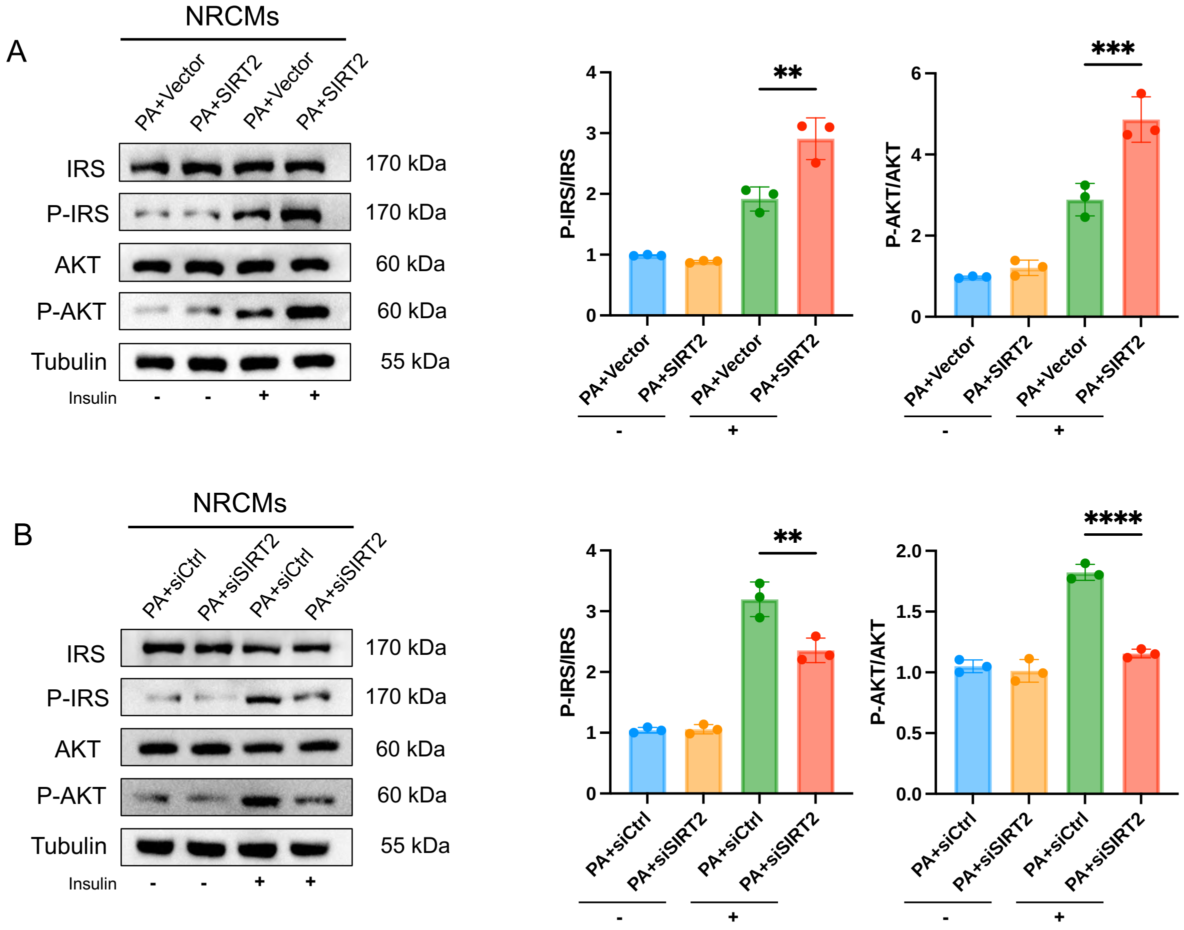

Supplementary Figure 9. Overexpression/deficiency of SIRT2 improves/impaires PA-induced insulin resistance in NRCMs. (A-B) Protein levels of insulin receptor signaling pathway was measured after saline or insulin (100 nmol/L) used for 10 min. (n=3). Data were presented as the mean ± SD. **p<0.01; ***p<0.001; ****p<0.0001. Statistical analysis was performed by one-way ANOVA.

Figure S10

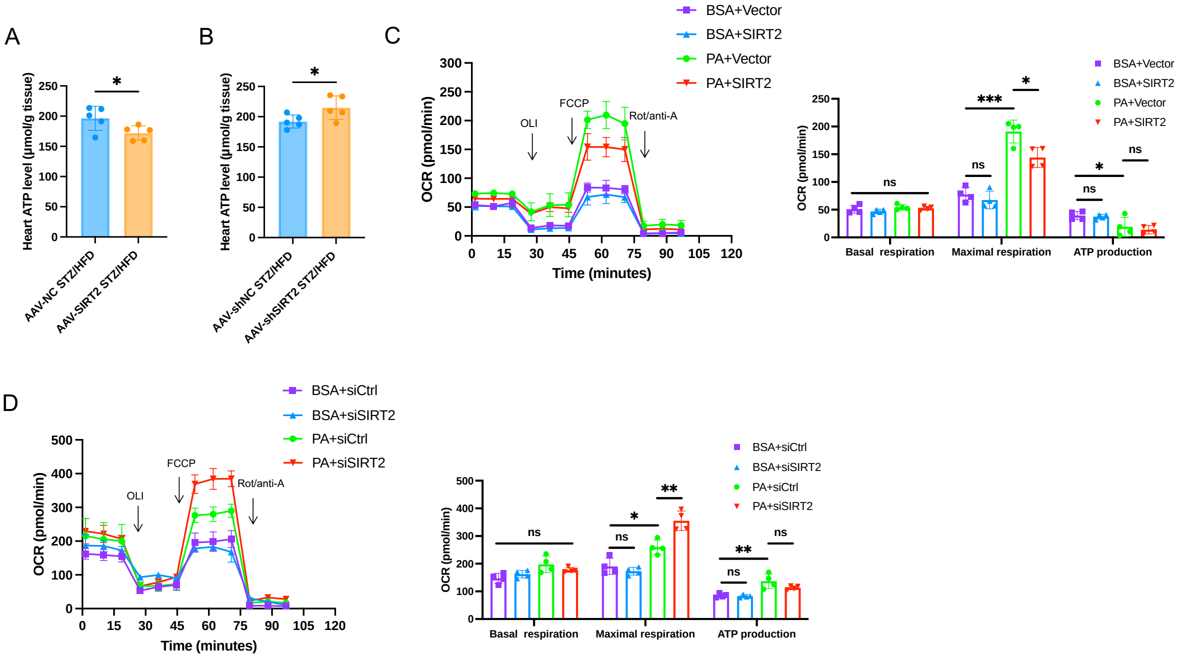

Supplementary Figure 10. Overexpression/deficiency of SIRT2 decreases/increases ATP and OCR levels. (A-B) Representative the heart ATP levels in mice (n=5). **(C-D)** OCR of AC16 cells was examined via the Seahorse assay (n=4). Data were presented as the mean ± SD. *p<0.05; **p<0.01; ***p<0.001; ns indicated no significance. Statistical analysis was performed by one-way ANOVA.

Figure S11

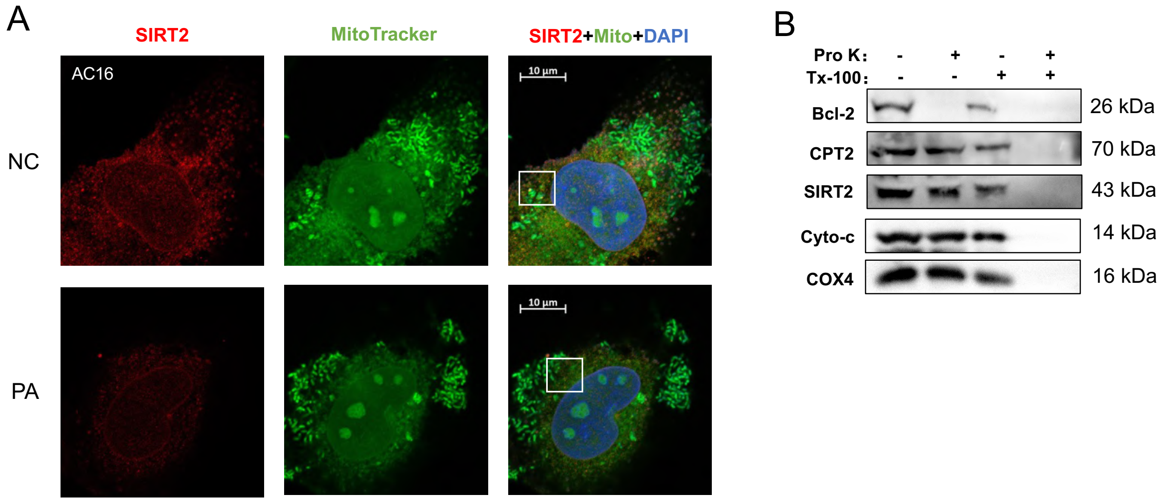

Supplementary Figure 11. SIRT2 is located in the mitochondria. (A) AC16 cells were stained with MitoTracker as well as SIRT2 and DAPI, and representative IF images were shown. Scale bar: 10 μm. **(B)** Mitochondria (100 μg per treatment) from AC16 cells were suspended in buffer with or without Triton X-100 (Tx-100) and proteinase K (Pro K). Samples were analyzed by western blotting. Bcl-2 (outer mitochondrial membrane), CPT2 (inner mitochondrial membrane), cytochrome C (intermembrane space), and COX4 (matrix) were used as markers.

Figure S12

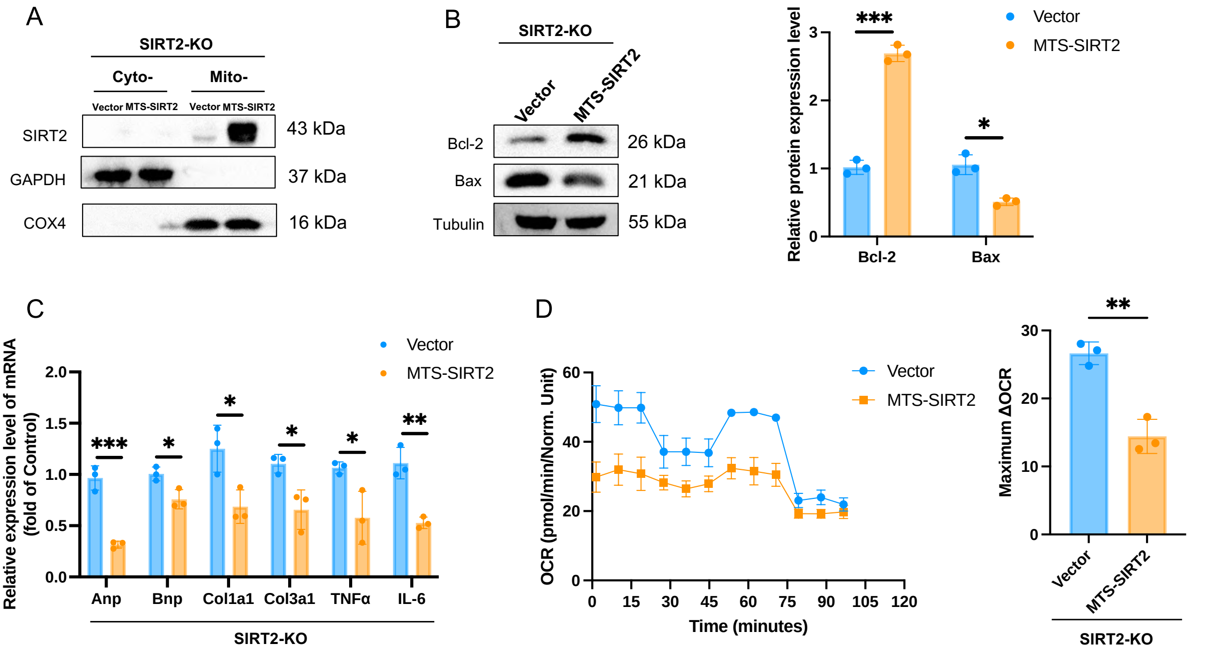

Supplementary Figure 12. Mitochondrial SIRT2 improves DCM mice. (A) Western blotting analysis of the mitochondria expression of SIRT2-MTS in AC16 SIRT2 knockout (KO) cells. **(B)** Protein levels of apoptosis gene Bax and anti-apoptosis gene Bcl2 in AC16 cells. **(C)** Hypertrophic genes and fibrotic genes mRNA expression in AC16 cells tested by qRT-PCR. **(D)** FAO of AC16 cells was examined via the Seahorse assay. (n=3). Data were presented as the mean ± SD. *p<0.05; **p<0.01; ***p<0.001; n.s indicated no significance. Statistical analysis was performed by unpaired Student's t test.

Figure S13

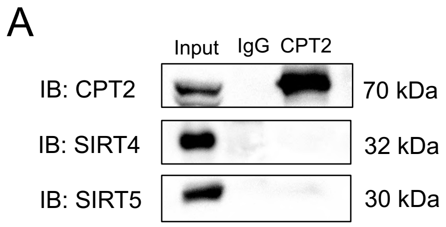

Supplementary Figure 13. (A) Coimmunoprecipitation analysis of SIRT4, SIRT5, and CPT2 in AC16 cells.

Figure S14

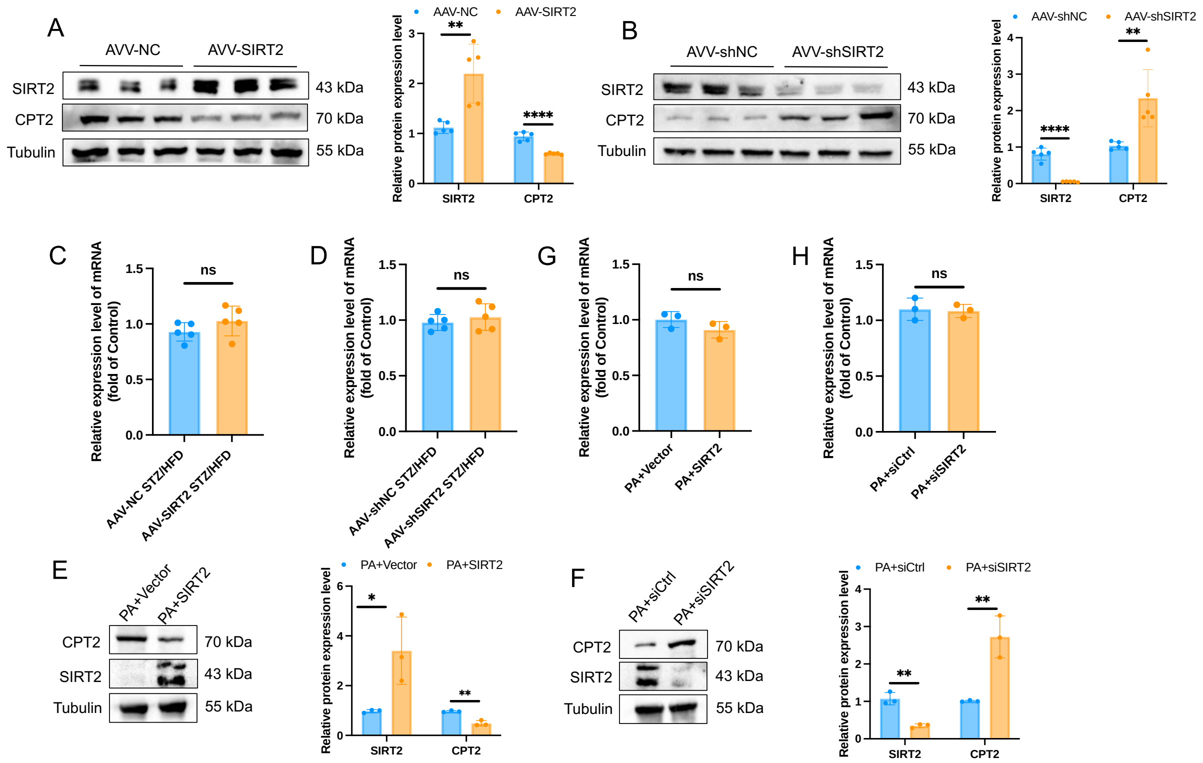

Supplementary Figure 14. SIRT2 negatively regulates the protein expression of CPT2. (A-B) SIRT2 and CPT2 protein expression levels in heart from AAV-NC or AAV-SIRT2 mice (A) and AAV-shNC or AAV-shSIRT2 mice (B) after STZ/HFD feeding for 20 weeks (n=5). **(C-D)** Representative mRNA levels of CPT2 in heart tissues (n=5). **(E-F)** SIRT2 and CPT2 protein expression levels in SIRT2 overexpressed (E) and downregulated (F) AC16 cells (n=3). **(G-H)** Representative mRNA levels of CPT2 in AC16 cells (n=3). Data were presented as the mean ± SD. *p<0.05; **p<0.01; ****p<0.0001; n.s indicated no significance. Statistical analysis was performed by unpaired Student's t test.

Figure S15

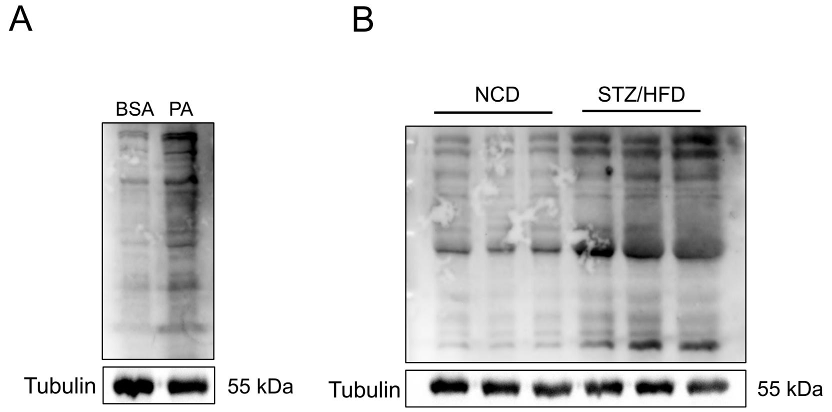

Supplementary Figure 15. Total protein acetylation levels. (A) Western blotting analysis of total protein acetylation levels in PA-treated cardiomyocytes (n=3). **(B)** Western blotting analysis of total protein acetylation levels in NCD and STZ/HFD mice (n=5).

Figure S16

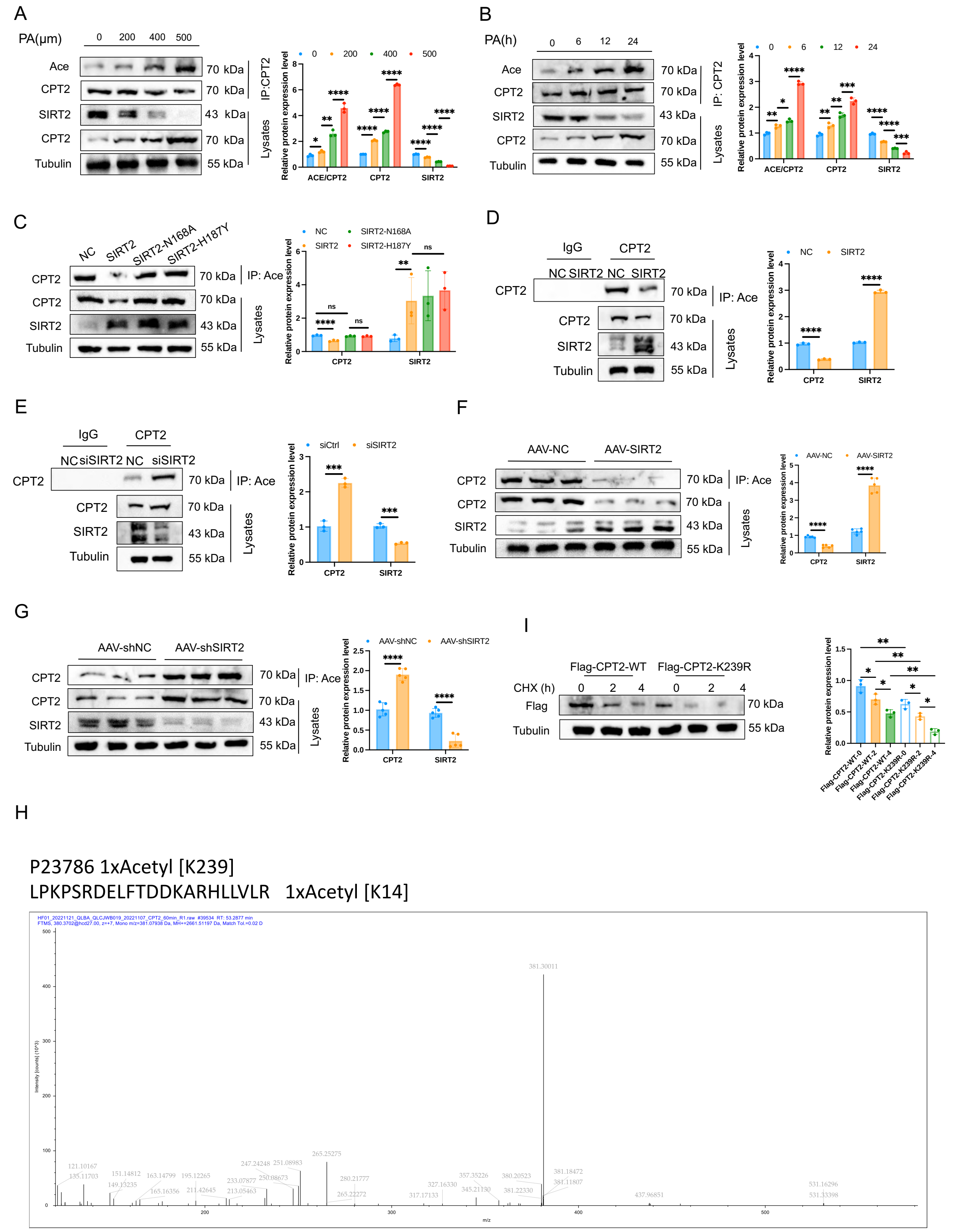

Supplementary Figure 16. Cardiac mitochondrial SIRT2 deacetylates CPT2. (A) Co-IP experiments of AC16 cells treated with PA at the concentration of 0 μM, 200 μM, 400 μM and 500 μM for 24 h, using an anti-CPT2 antibody for IP and anti-acetyl-lysine antibodies for immunoblotting (n=3). **(B)** Co-IP experiments of AC16 cells treated with 0.5 mM PA for the indicated time, using an anti-CPT2 antibody for IP and anti-acetyl-lysine antibodies for

immunoblotting (n=3). **(C)** Co-IP experiments of AC16 cells expressing NC, SIRT2 or mutant plasmids (SIRT2-N168A, SIRT2-H187Y), using an anti-acetyl-lysine antibody for IP and anti-CPT2 antibodies for immunoblotting (n=3). **(D)** Co-IP experiments of AC16 cells overexpressing NC or SIRT2 plasmids, using an anti-acetyl-lysine antibody for IP and anti-CPT2 antibodies for immunoblotting (n=3). **(E)** Co-IP experiments of AC16 cells transfected with siCtrl or siSIRT2, using an anti-acetyl-lysine antibody for IP and anti-CPT2 antibodies for immunoblotting (n=3). **(F)** Co-IP assay of cardiomyocytes s from AAV-NC or AAV-SIRT2 mice, using an anti-acetyl-lysine antibody for IP and anti-CPT2 antibodies for immunoblotting (n=5). **(G)** Co-IP assay of cardiomyocytes from AAV-shNC or AAV-shSIRT2 mice, using an anti-acetyl-lysine antibody for IP and anti-CPT2 antibodies for immunoblotting (n=5). **(H)** The mass spectrometry identification map of CPT2 K239 acetylation in human AC16 cells. **(I)** CPT2 protein stability time course of overexpressed CPT2-WT and K239R mutants after treatment with 100μg/mL CHX in HEK293T cells (n=3). Data were presented as the mean ± SD. *p<0.05; **p<0.01; ***p<0.001; ****p<0.0001;ns indicated no significance. Statistical analysis was performed by one-way ANOVA and unpaired Student’s t test.

Figure S17

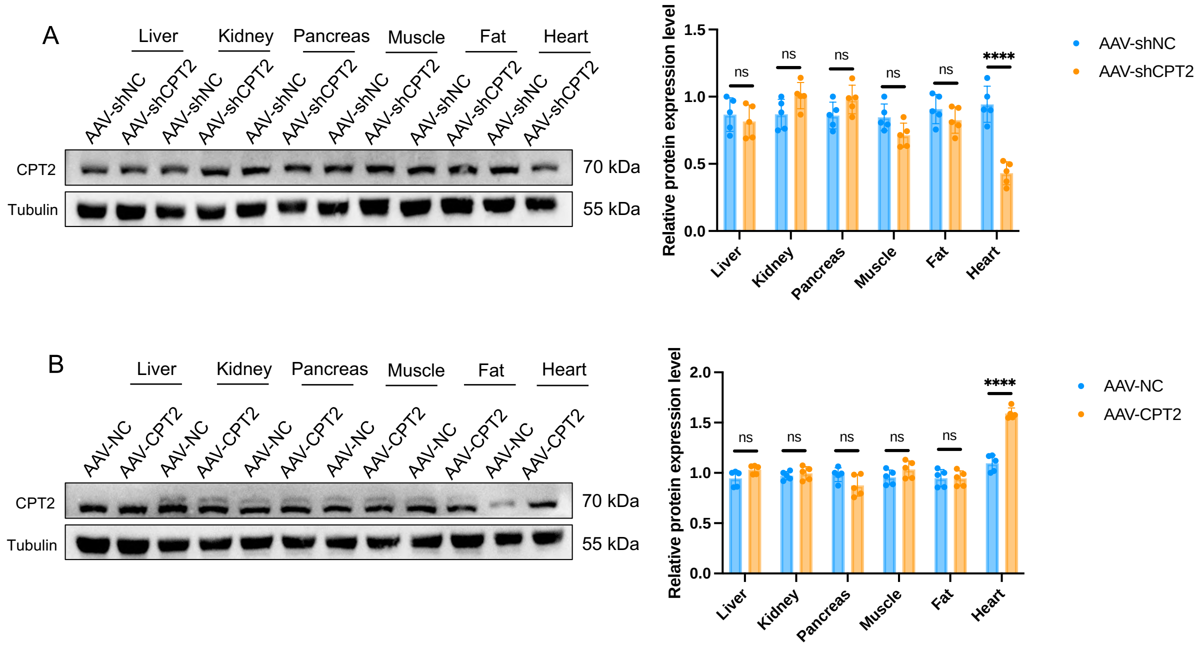

Supplementary Figure 17. Cardiac-specific CPT2 deficiency/overexpression male mice are established by AAV. (A-B) The protein expression of CPT2 in the liver, kidney, pancreas, muscle, fat, and heart (n=5). Data were presented as the mean ± SD. ****p<0.0001; ns indicated no significance. Statistical analysis was performed unpaired Student's t test.

Figure S18

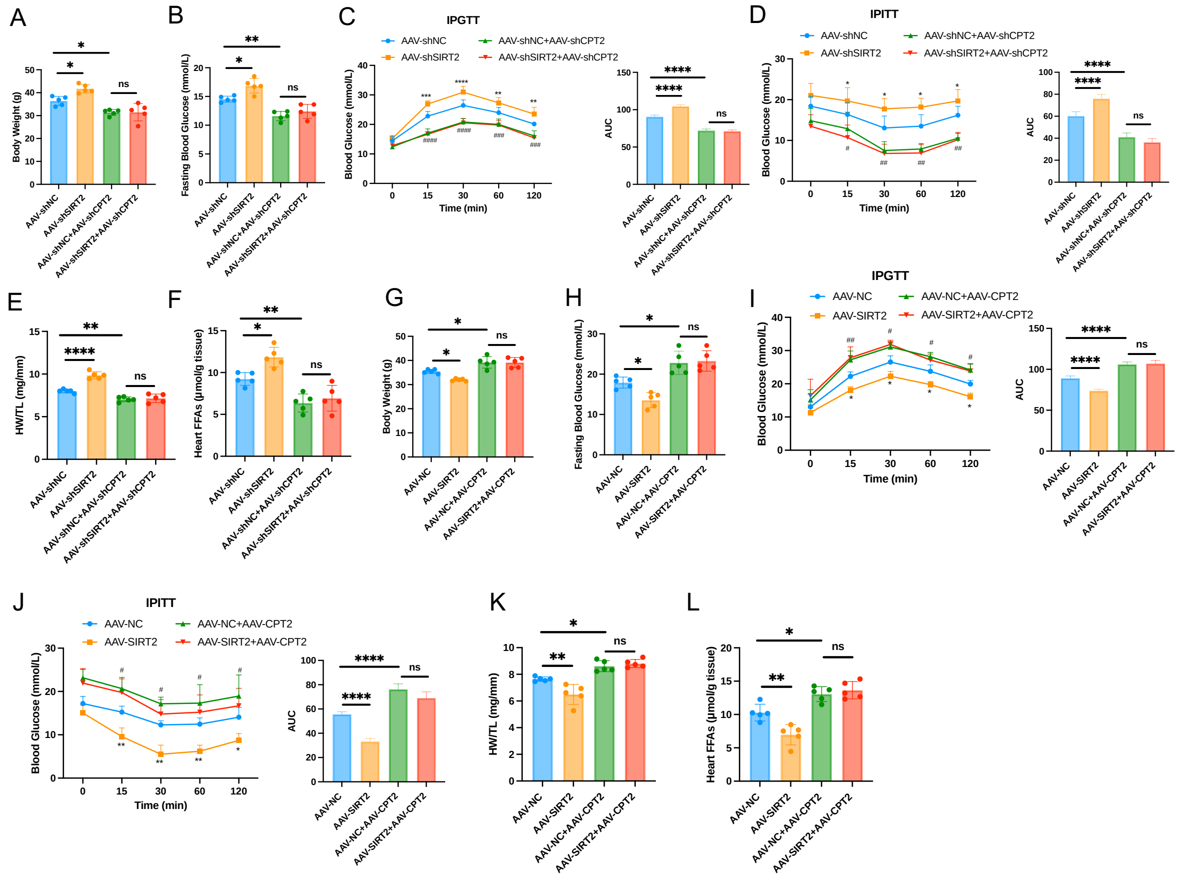

Supplementary Figure 18. Deficiency/Overexpression of SIRT2 and CPT2 improves/aggravates peripheral glycolipid metabolism and heart FFAs in diabetic cardiomyopathy male mice. (A-B) Representative body weight and fasting blood glucose in AAV-shNC, AAV-shSIRT2, AAV-shNC+AAV-shCPT2, and AAV-shSIRT2+AAV-shCPT2 mice. **(C-D)** IPGTT/IPITT was performed after feeding for 20 weeks. Blood glucose levels were examined at the indicated times post-glucose/insulin injection. The area under the curve was calculated. * represented AAV-shSIRT2 and AAV-shNC, $^{\#}$ represented AAV-shNC+AAV-shCPT2 and AAV-shNC. **(E)** Representative the HW/TL in mice. **(F)** Representative the heart FFAs levels in mice. **(G-H)** Representative body weight and fasting blood glucose in AAV-NC, AAV-SIRT2, AAV-NC+AAV-CPT2, and AAV-SIRT2+ AAV-CPT2 STZ/HFD mice. **(I-J)** IPGTT/IPITT was performed in 20-week-old mice. Blood glucose levels were examined at the indicated times post-glucose/insulin injection. The area under the curve was calculated. * represented AAV-SIRT2 and AAV-NC, $^{\#}$ represented AAV-NC+AAV-CPT2 and AAV-NC. **(K)** Representative the HW/TL in mice. **(L)** Representative the heart FFAs levels in mice. (n=5). Data were presented as the mean ± SD. *p<0.05; **p<0.01; ***p<0.001; ****p<0.0001; $^{\#}$p<0.05; $^{\#\#}$p<0.01; $^{\#\#\#}$p<0.001; $^{\#\#\#\#}$p<0.0001; ns indicated no significance. Statistical analysis was performed by one-way ANOVA.

Figure S19

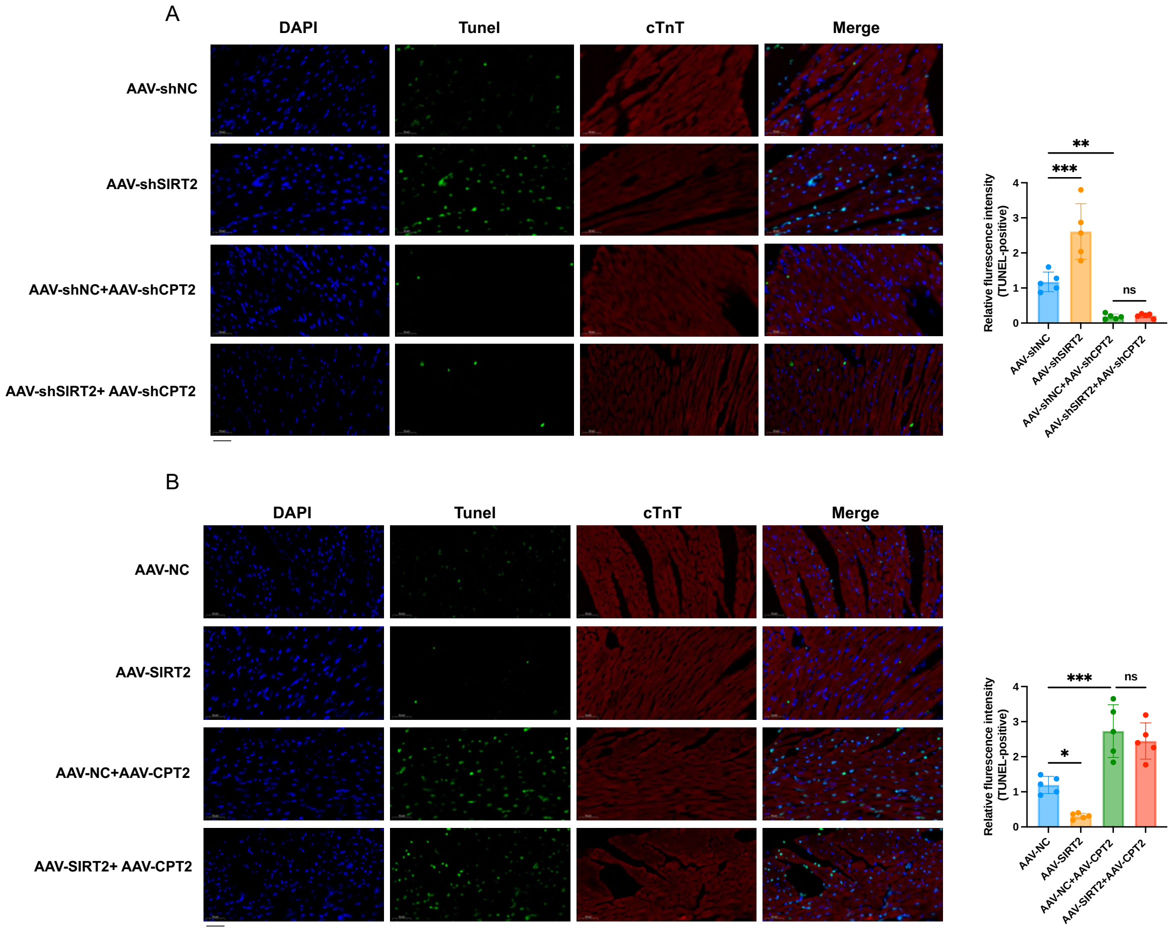

Supplementary Figure 19. Deficiency/Overexpression of SIRT2 and CPT2 improves/aggravates cell apoptosis in diabetic cardiomyopathy male mice. (A-B) TUNEL assay in mouse heart tissues co-staining with cTnT (n=5). Scale bar, 50 μm. Data were presented as the mean ± SD. *p<0.05; **p<0.01; ***p<0.001; ns indicated no significance. Statistical analysis was performed by one-way ANOVA.

Figure S20

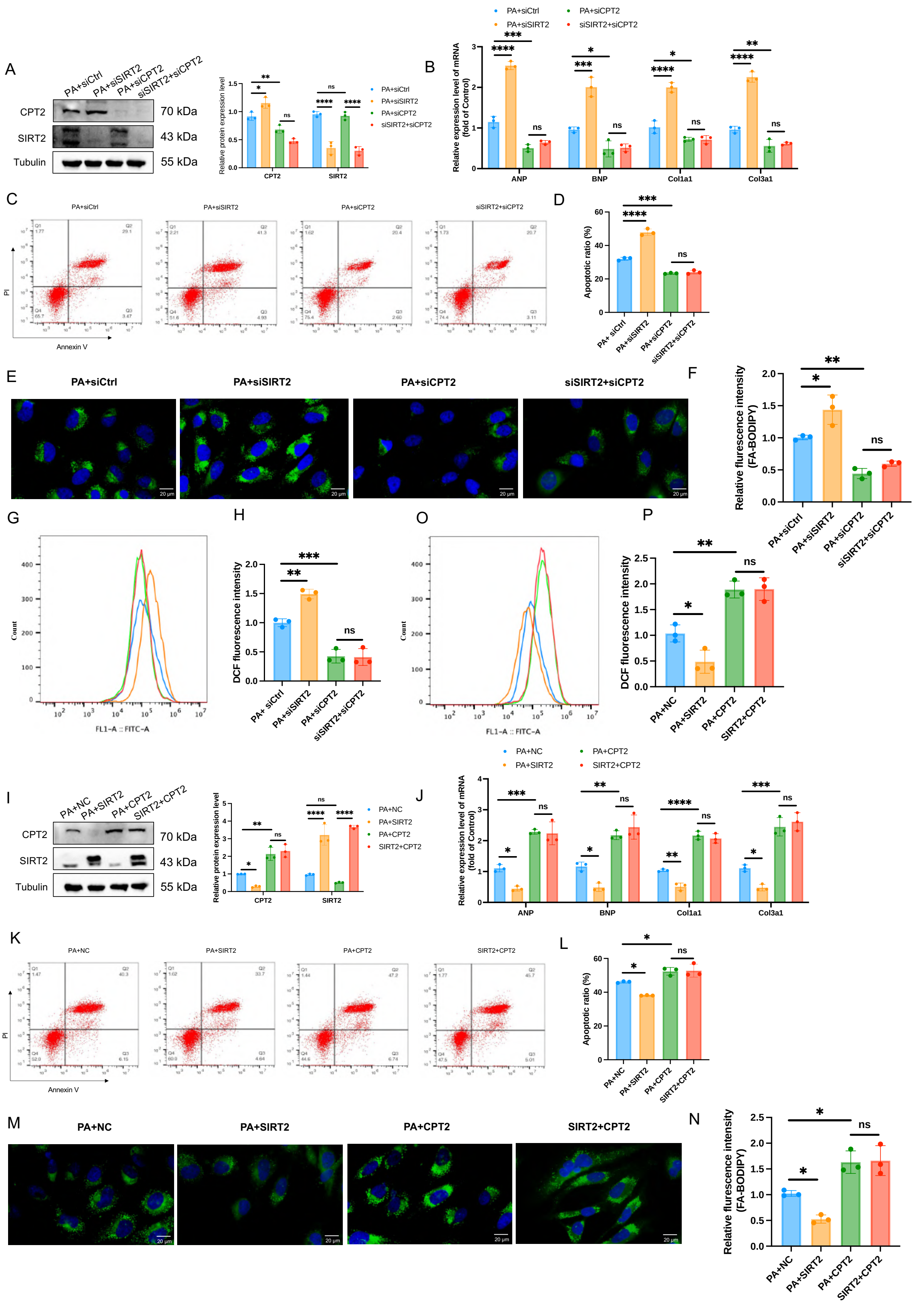

Supplementary Figure 20. Downregulation/upregulation of CPT2 promotes/inhibites the function of SIRT2-deficiency in PA-induced cardiomyocytes. (A) siCtrl and siSIRT2 were

used to interfere with intracellular SIRT2, followed by siCPT2 and PA treated for 24 h. The protein expression of SIRT2 and CPT2 in AC16 cells. **(B)** Hypertrophic genes and fibrotic genes mRNA expression in AC16 cells tested by qRT-PCR. **(C-D)** Apoptosis of AC16 cells was measured by flow cytometry with Annexin V and PI staining. **(E-F)** Representative images and quantitative analysis of BODIBY 493/503 fluorescent dye staining of neutral lipid level in cells. Scale bars, 20μm. **(G-H)** ROS generation in cells was examined by flow cytometry with DHE probe treatment. **(I)** Vector and SIRT2 plasmids were used to increase intracellular SIRT2, followed by CPT2 plasmids and PA treated for 24 h. The protein expression of SIRT2 and CPT2 in AC16 cells. **(J)** Hypertrophic genes and fibrotic genes mRNA expression in AC16 cells tested by qRT-PCR. **(K-L)** Apoptosis of AC16 cells was measured by flow cytometry with Annexin V and PI staining. **(M-N)** Representative images and quantitative analysis of BODIBY 493/503 fluorescent dye staining of neutral lipid level in cells. Scale bars, 20μm. **(O-P)** ROS generation in cells was examined by flow cytometry with DHE probe treatment. (n=3). Data were presented as the mean ± SD. *p<0.05; **p<0.01; ***p<0.001; ****p<0.0001; ns indicated no significance. Statistical analysis was performed by one-way ANOVA.

Figure S21

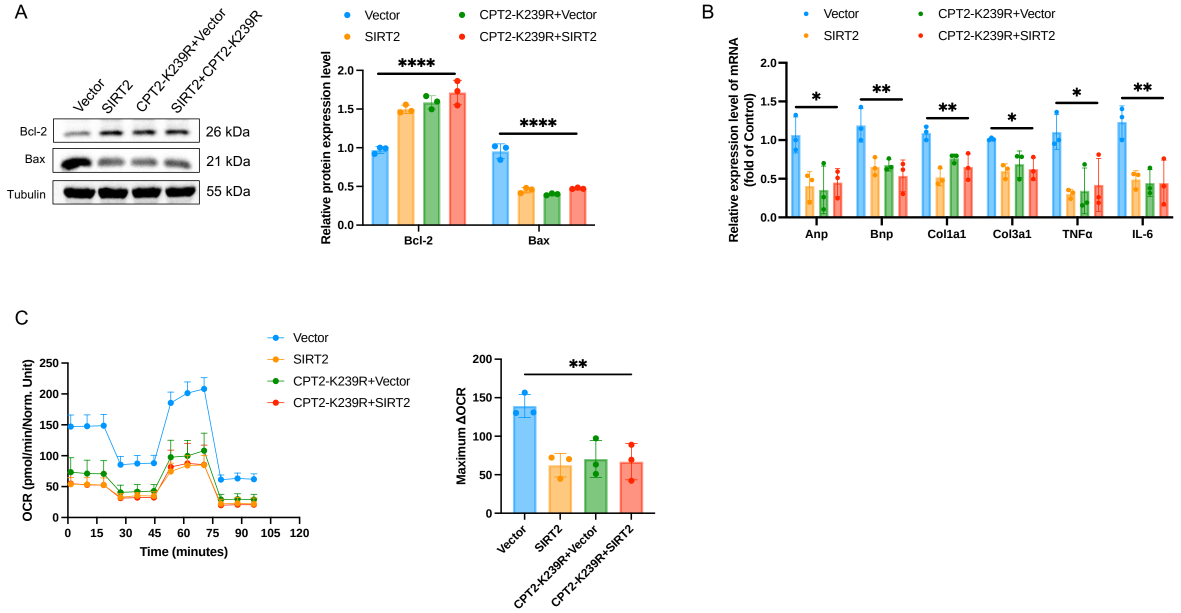

Supplementary Figure 21. SIRT2 improves DCM through Lys239 deacetylation of CPT2. (A) Protein levels of apoptosis protein Bax and anti-apoptosis Bcl2 in AC16 cells. **(B)** Hypertrophic genes (Anp, Bnp) and fibrotic genes (Col1a1, Col3a1) mRNA expression in AC16 cells tested by qRT-PCR. **(C)** FAO of AC16 cells was examined via the Seahorse assay. (n=3). Data were presented as the mean ± SD. *p<0.05; **p<0.01; ****p<0.0001; ns indicated no significance. Statistical analysis was performed by one-way ANOVA.

Supplementary Tables

Table S1. Primers for plasmids construction.

Gene	Forward	Reverse
HA-SIRT2	AGCTTTGTTTAAACATGGCAGAG CCAGACCCCTCT	CCGGAATTCTTCACTGGGGTTTC TCCCTCTC
SIRT2-N168A	CTGCGCTGCTACACGCAGGCCAT AGATACCCT	GCCTGCGTGTAGCAGCGCAGGA GTAGCCCCTT
SIRT2-H187Y	GAGGACTTGGTGGAGGCGGCCG GCACCTTCTA	GCCGCCTCCACCAAGTCCTCCTG TTCCAGCCC
Flag-CPT2	AACCGTCAGATCCGCTAGCCACC ATGGTGCCCCGC	GGTCTTTGTAGTCGGATCCACTT TTGATGGATTTGCCTTC
CPT2-K457R	GAATTCCTGAGGAAGCAAAAGC TGAGCCCTGA	TGCTTCCTCAGGAATTCTTTGCC TCCTCTCTG
CPT2-K453R	AGAGGAGGCAGGGAATTCCTGA AGAAGCAAAAGCTG	AATTCCCTGCCTCCTCTCTGAAA CTGGACGCA
CPT2-K79R	GTTCAGGAGGACAGAACAATTTT GCAAGAGTTTTG	GTTCTGTCCTCCTGAACTGGCCA TCATTCAAG
CPT2-K239R	TTCACTGATGACAGGGCCAGACA CCTCCTGGTCCT	GCCCTGTCATCAGTGAAGAGTTC ATCCCGACT

Table S2. The antibodies were used in this study.

Antibody	Company	Catalog No.
SIRT2	Abcam	ab211033
SIRT2	Proteintech	66410-1-Ig
CPT2	Abcam	ab110293
CPT2	Proteintech	26555-1-AP
SIRT3	ABclonal	A12575
SIRT4	ABclonal	A20805
SIRT5	ABclonal	A23083
Cytochrome C	ABclonal	A4912
IRS	Abcam	ab46800
p-IRS	Cell Signaling Technology	3070
AKT	Abcam	ab8805
p-AKT	Cell Signaling Technology	9271
Bcl2	Abcam	ab182858
Bax	Cell Signaling Technology	2772
Ubiquitin	Abcam	ab134953
Flag	Cell Signaling Technology	14793
HA	Cell Signaling Technology	3724
Acetylated lysine	PTMBIO	PTM-101
Tubulin	Proteintech	10068-1-AP
HRP-goat anti-mouse	Proteintech	SA00001-1
HRP-goat anti-rabbit	Proteintech	SA00001-2

Table S3. Sequence of primers for PCR.

Gene	Forward	Reverse
**Mouse**		
β-Actin	GTGACGTTGACATCCGTAAAGA	GCCGGACTCATCGTACTCC
Anp	TCTTCCTCGTCTTGGCCTTT	CCAGGTGGTCTAGCAGGTTC
Bnp	TGGGAGGTCACTCCTATCCT	GGCCATTTCCTCCGACTTT
Col1a1	CTGGCGGTTCAGGTCCAAT	TTCCAGGCAATCCACGAGC
Col3a1	TGAATGGTGGTTTTCAGTTCAG	GATCCCATCAGCTTCAGAGACT
Sirt2	GCGGGTATCCCTGACTTCC	CGTGTCTATGTTCTGCGTGTAG
TNF-α	CGTCAGCCGATTTGCTATCT	CGGACTCCGCAAAGTCTAAG
IL-6	AGTTGCCTTCTTGGGACTGA	TCCACGATTTCCCAGAGAAC
SIRT3	CTGCTACTCATTCTTGGGACC	GGACCACATCTTTCCTTCGAG
SIRT4	GATTGACTTTCAGGCCGACAA	GCGGCACAAATAACCCCGA
SIRT5	ATCGCAAGGCTGGCACCAAGAA	CTAAAGCTGGGCAGATCGGACT
**Rat**		
β-Actin	*ACAGCAACAGGGTGGTGGAC*	*ACAGCAACAGGGTGGTGGAC*
Anp	*GAAGATGCCGGTAGAAGATGAG*	*AGAGCCCTCAGTTTGCTTTTC*
Bnp	*GGTGCTGCCCCAGATGATT*	*CTGGAGACTGGCTAGGACTTC*
Col1a1	*CACTGCAAGAACAGCGTAGC*	*AGTTCCGGTGTGACTCGTG*
Col3a1	*AGGTCCAGGGATACGGGGTA*	*CAGGGAAACCCATGACACCA*
**Human**		
β-Actin	*CATGTACGTTGCTATCCAGGC*	*CTCCTTAATGTCACGCACGAT*
Anp	*CACCGTGAGCTTCCTCCTTT*	*CCAAATGGTCCAGCAAATTCTTG*
Bnp	*TGCTCTTCTTGCATCTGGCTT*	*ATGGTTGCGCTGCTCCTGTAA*
Col1a1	*CCCCGAGGCTCTGAAGGT*	*GCAATACCAGGAGCACCATTG*
Col3a1	*CCTGAAGCTGATGGGGTCAA*	*CCCCAGTGTGTTTCGTGCAA*
